# Human pluripotent stem cell-derived neuroepithelial cells develop into an organizer for optic tectum formation in the chicken diencephalon

**DOI:** 10.3389/fcell.2026.1812038

**Published:** 2026-06-01

**Authors:** Meghdad Yeganeh, Mostafa Najar-Asl, Razieh Karamzadeh, Shiva Nemati, Saeed Yakhkeshi, Azimeh Akhlaghpour, Stefan Guenther, Somayeh Naderi, Ebrahim Shahbazi, Sepideh Mollamohammadi, Susan Simorgh, Seyedeh-Nafiseh Hassani, Amir Shojaei, Javad Mirnajafizadeh, Mohammad-Reza Abolghasemi Dehaqani, Majid Nili Ahmadabadi, Ruijin Huang, Koorosh Shahpasand, Shin-Ichi Hisanaga, Thomas Braun, Hossein Baharvand

**Affiliations:** 1 Department of Stem Cells and Developmental Biology, Cell Science Research Center, Royan Institute for Stem Cell Biology and Technology, ACECR, Tehran, Iran; 2 School of Biotechnology, College of Science, University of Tehran, Tehran, Iran; 3 Animal Biotechnology Research Institute, Agricultural Research, Education and Extension Organization (AREEO), Agricultural Biotechnology Research Institute of Iran (ABRII), Rasht, Iran; 4 Max-Planck Institute for Heart and Lung Research, Department of Cardiac Development and Remodelling, Bad Nauheim, Germany; 5 Department of Physiology, Faculty of Medical Sciences, Tarbiat Modares University, Tehran, Iran; 6 Cognitive Systems Laboratory, Control and Intelligent Processing Center of Excellence (CIPCE), School of Electrical and Computer Engineering, College of Engineering, University of Tehran, Tehran, Iran; 7 Department of Brain and Cognitive Sciences, Cell Science Research Center, Royan Institute for Stem Cell Biology and Technology, ACECR, Tehran, Iran; 8 Institute of Anatomy, University of Bonn, Bonn, Germany; 9 Department of Biological Sciences, Graduate School of Science, Tokyo Metropolitan University, Hachioji, Tokyo, Japan; 10 Department of Dementia and Higher Brain Function, Tokyo Metropolitan Institute of Medical Science, Setagaya, Tokyo, Japan; 11 Department of Developmental Biology, School of Basic Sciences and Advanced Technologies in Biology, University of Science and Culture, Tehran, Iran

**Keywords:** brain development, isthmic organizer, neuroepithelial cells, optic tectum, pluripotent stem cells

## Abstract

Human brain development critically depends on the formation of secondary organizers, which determine specification of neural territories along the antero-posterior axis. Studies on human secondary organizers have proven to be difficult due to technical limitations and ethical concerns. Here, using interspecies transplantations we demonstrate that neuroepithelial cells (NECs) derived from human pluripotent stem cells (hPSCs) acquire properties of the isthmic organizer (IsO) when transplanted into the HH11 chicken anterior diencephalon, driving formation of a fully laminated optic tectum. hPSCs-derived NECs initially do not express the whole range of characteristic IsO molecules, but develop these features after transplantation and interaction within host tissue. We show that engineered human NECs act as dominant IsOs, overriding local signals in the chicken diencephalon to form a mesencephalic architecture, the optic tectum. The generation of IsOs from hPSCs offers new opportunities to study the pathogenesis of brain defects caused by malfunctions of the IsO.

## Introduction

Generation of neurons and glia cells from human pluripotent stem cells (hPSCs) has become a routine procedure, raising hopes for clinical applications, e.g., for the treatment of neurodegenerative disorders such as Parkinson’s disease and Alzheimer’s disease. To achieve this goal either a specific type of neurons (e.g., dopaminergic neurons for treatment of Parkinson’s disease) or neural stem cells are produced, which can replace lost or damaged cells. Equally important is the use of hPSCs for establishing new models for neurological diseases, which recently evolved into generation of human brain organoids (Di Lullo and Kriegstein, 2017). Brain organoids provide the opportunity to model certain aspects of human brain development and investigate mechanisms of intrinsic patterning within organoids that allow formation of diverse brain regions (Sidhaye and Knoblich, 2021). Exposure of brain organoids to patterning factors, fusion of differentially patterned organoids or co-culture of brain organoids with other cell types have been used to improve organoid methodology. In principle, it is also feasible to inject distinct hPSC-derived neuroepithelial cells (NECs) into brain to explore potential morphogenetic effects (Reubinoff et al., 2001; Zhang et al., 2001). However, surprisingly little attention has been paid to the differentiation of hPSCs into neuroepithelial cells with characteristics of secondary organizers.

Secondary organizers are local signaling centers in the neuroepithelium, which specify different neural territories along the antero-posterior axis during neural plate and neural tube stages (Echevarria et al., 2003; Kiecker and Lumsden, 2012). A prominent example for a secondary organizer is the isthmic organizer (IsO) at the midbrain-hindbrain boundary (Martinez, 2001; Nakamura et al., 2005; Nakamura et al., 2008). The IsO controls regionalization of the midbrain and anterior hindbrain and is critical for specification of cerebellar, isthmic, and mesencephalic territories. The crucial morphogenetic role of the chicken IsO for the development of adjacent regions has also been demonstrated by ectopic transplantation to rostral regions of the neural tube (for review see Vieira et al. (2010)). The IsO emerges between the caudal and rostral boundaries of *Otx2* and *Gbx2* activities and is characterized by expression of *Lmx1b* and *Fgf8* (Adams et al., 2000; Chandel and Hornblad, 2024 *)*. Deletion of *Wnt1, Pax2, En1, Gbx2* or *Fgf8* in mice prevents development of isthmo-cerebellar structures (Hidalgo-Sanchez et al., 2022). Fgf8 is not only essential for formation of the IsO but also critical for IsO activity, since ectopic expression of Fgf8 protein induces IsO-characteristic in the diencephalic caudal prosomeres (p1-p2), the midbrain, and the hindbrain (Canning et al., 2007). Distortion of IsO formation is associated with malformations of the human CNS (Barkovich et al., 2009), necessitating further research in the underlying mechanisms.

Xenotransplantation of hPSC-NECs, neurons, or glia cells into chicken embryos represents an alternative approach to brain organoids for studying effects of human cells on brain development. In contrast to organoids, developing chicken brains offer easy accessibility, always form the same architecture at the same location and time, and contain all cell types required to generate a fully functional brain. Thus, developing chicken brains can be employed as an almost ideal *in vivo* incubator to study properties of human neuroepithelial secondary organizers. Transplantation of cells derived from human embryonic stem cells (hESCs) and human induced pluripotent stem cells (hiPSCs) has been used in the past to understand mechanisms of organ development (Martyn et al., 2018; Nguyen et al., 2018). Likewise, IsO-like cells have been generated from hESCs before, but the ability of such cells to exert secondary organizer function *in vivo* has not been tested (Lee et al., 2018).

Here we found that hPSC-NECs transplanted into the most anterior diencephalic area of early chicken embryo induce an ectopic, fully laminated optic tectum (TeO) with a 15-layer architecture. Strikingly, the TeO strongly resembled a prominent part of midbrain that is responsible for receiving and processing the visual input from retinal ganglion cells (Wylie et al., 2009; Verhaal and Luksch, 2013; Lever et al., 2014). RNA-seq analysis revealed expression of IsO genes in transplanted human cells and demonstrated that the newly induced tectum in the diencephalon resembles the naturally developed chicken tectum but not the cerebral cortex. Furthermore, increased expression of doublecortin (DCX), reelin, and genes involved in ECM-receptor interaction and cell adhesion indicated enhanced migration of newborn neurons to form a laminar architecture.

## Materials and methods

### Ethics statement

This study was conducted according to protocols approved by the Royan institutional review board and institutional ethical committee (No: IR. ACECR.Royan.Rec.1395.132). Royan ethical committee is composed of members with scientific, legal, clergyman and bioethical expertise. No chicken-human chimeras were allowed to hatch.

### Culture of hPSCs

The hESC line RH6 (Baharvand et al., 2006), the hiPSC lines RiPSC4 (Totonchi et al., 2010), and the iPSC line CiPSC (Xcellscience, IP-001-ZIV8-A-1V) were used in the study. The lines were checked routinely for *mycoplasma* contamination and cultured in feeder free conditions. Cells were maintained in a humidified incubator at 37 °C and 5% CO2 condition on Matrigel (Sigma-Aldrich, E1270)-coated plates in hPSC medium.

Cells were cultured in Dulbecco’s modified Eagle’s medium (DMEM)/F12 (Gibco, 21331-020) supplemented with 20% knockout serum replacement (KSR, Gibco, 10828-028), 2 mM L-glutamine (Gibco, 25030-024), 0.1 mM non-essential amino acids (Gibco, 11140-035), 1X ITS-G (1 mg/mL insulin, 0.55 mg/mL transferrin, 0.00067 mg/mL sodium selenite; Gibco, 41400-045), 0.1 mM β-mercaptoethanol (Sigma-Aldrich, M7522), 100 U/mL penicillin, 100 μg/mL streptomycin (Gibco, 15140122) and 100 ng/mL bFGF (Royan Biotech, RP-1101-50).

For passaging every 7 days, cells were rinsed with Ca^2+^/Mg^2+^-free phosphate-buffered saline (PBS; Gibco, 21600-051) before enzymatic dissociation (trypsin solution (2.5% wt/vol), KSR, CaCl2 (100 mM) and PBS) at 37 °C for 5–7 min. The ROCK inhibitor, Y-27632 (Sigma-Aldrich, 129830-38-2) was added to the culture medium at a final concentration of 10 µM 1 h before dissociation. The medium was changed every day.

### Cell transfection

To generate the CMV early enhancer/chicken beta actin promoter–human recombinant green fluorescent protein human embryonic stem cell (CAG-hrGFP hESC) line, 10 µM ROCK inhibitor, Y-27632 was added to the medium. hESCs RH6 (P18) were dissociated with TrypLE (Gibco, 12605-028) and 4 ×10^6^ cells resuspended in 700 µL Opti-MEM (Gibco, 11058-021).

Electroporation was performed using 14 µg of each TALEN-encoding vectors including AAVS1-CAG-hrGFP (Addgene, 52344), hAAVS1 TALEN right (Addgene, 52342) and hAAVS1 TALEN left (Addgene, 52341) (Gene Pulser Xcell System, Bio-Rad, 250 V, 500 mF, 0.4-Cm cuvettes). Next, 1 ×10^6^ transfected cells were seeded in a 6 cm^2^ tissue culture dish coated with puromycin-resistant mouse embryonic fibroblast feeder, F-DR4, in hESC medium supplemented with Y-27632 for the first 24 h. After 3 days, cells were treated with 1 μg/mL puromycin for selecting single resistant clones.

### Differentiation of hPSCs to NECs

Feeder-free, high density monolayer hPSCs grown for 7 days were induced to differentiate to NECs for 3 days in Dulbecco’s Modified Eagle Medium/Nutrient Mixture F-12 (DMEM/F12, Gibco, 21331020), 5% KSR (Gibco, 10828028), 2 mM Glutamax (Gibco, 25030081), 1% MEM-non-essential amino acids (NEAA, Gibco, 11140050), and 1% N2 (Gibco, 17502048) with 100 ng/mL noggin (R&D, 6057-NG-100, a BMP inhibitor), 3 μM SB431542 (Cyman, 13031, a TGFβ inhibitor), 0.5 μM LDN193189 (Stemgent, 04-0074, a BMP inhibitor), 50 ng/mL sonic hedgehog (Shh, R&D, 1845-SH-025), 2.5 μM purmorphamine (Stemgent, 04-0009, a Shh agonist), 3 μM CHIR99021 (CHIR, Stemgent, 04-0004-10, a GSK3β inhibitor), and 1 μM retinoic acid (RA, Sigma-Aldrich, R2625). The medium was changed every day.

We also used an alternative differentiation protocol for generation of NECs that was published before (Tchieu et al., 2017). Two different media were used in this protocol: KSR differentiation medium and N2 medium. 1L of KSR differentiation medium consist of 820 mL Knockout DMEM (1X) medium (Gibco, 10829018), 150 mL KSR, 10 mL penicillin-streptomycin, 10 mL Glutamax 200 mM, 10 mL MEM-NEAA 100x, and 1 mL 2-mercaptoethanol 1000x. 1 L of N2 medium consists of 0.5 L DMEM, 0.5L F12, 1 mL 2-mercaptoethanol 1,000x, 2.0 g Sodium bicarbonate (Sigma-aldrich, S5761), 1.56 g D-(+)- Glucose (Sigma-Aldrich, G8769), and 20 µL progesterone (Stock: dissolve 0.032 g Progesterone (Sigma-Aldrich, P6149) in 100 mL 100% ethanol), with 10 mL N2 supplement 100x. The small molecules LDN (500 nM), SB (10 µM), and XAV939, a WNT pathway inhibitor (XAV, Tocris, 3748/10, 5 µM) were added from day 0–5 to the medium, followed by treatment until day 12 in the absence of XAV. Cells were cultured in 100% KSR differentiation medium until day 4. Afterwards the 100% KSR differentiation medium was gradually reduced to 25% every other day and replaced with N2 medium until day 10, at which cells were placed in N2 medium.

### Immunofluorescence staining of cultured cells

For immunofluorescence analysis, cells were fixed in 4% paraformaldehyde (PFA, Sigma-Aldrich) for 20 min at room temperature (RT, 23 °C–25 °C), permeabilized with 0.1% Triton X-100 for 10 min (except for staining for surface markers), blocked in 10% of primary antibody host serum in PBS for 1 h at RT, and incubated overnight with primary antibodies at 4 °C (1 hour incubation at RT for staining for surface markers). Afterwards, cells were washed and incubated with secondary antibodies for 45 min in an incubator at 37 °C (except directly conjugated antibodies). The following primary antibodies were used: mouse anti-NESTIN (Chemicon, MAB5326, 1:100), rabbit anti-SOX2 (Abcam, Ab69893, 1:250), mouse anti-SOX1 (R&D system, MAB3369, 1:250), rabbit anti-SOX1 (Abcam, Ab22572, 1:250), mouse anti-NCAM (Abcam, Ab6123, 1:250), rat anti-CD133 (ebioscience, 11-1331-82, 1:250), and rabbit anti-PAX6 (Abcam, Ab195045, 1:250). Secondary antibodies were goat anti-mouse Alexa Fluor® 488 (Invitrogen, A11001, 1:400), goat anti-rabbit Alexa Fluor® 488 (Invitrogen, A11034, 1:500), goat anti-mouse Alexa Fluor® 568 (Invitrogen, A11004, 1:500) and goat anti-rat Alexa Fluor® 488 (Invitrogen, A11006, 1:500).

For negative controls, cells were only incubated cells with secondary antibodies after permeabilization. Nuclei were stained by incubating with 4, 6-diamidino-2-phenylindole (DAPI, 5 μg/mL, Sigma-Aldrich, 32,670) in PBS for 3 min at RT. The cells were analyzed with a fluorescent microscope IX71 (Olympus).

### Flow cytometry

Cells were dissociated with 0.05% trypsin-EDTA (Gibco-25300054)) and fixed in 4% PFA for 20 min at RT. Next, cells were washed and permeabilized (except for surface marker staining) with 0.1% Triton X-100 for 10 min at RT after which 10% of primary antibody host serum in PBS was used to block non-specific antibody binding. Subsequently, the cell suspension was incubated overnight at 4 °C with the primary antibody (1 hour at RT for staining for surface markers) diluted in PBS containing 0.1% FBS and 0.1% BSA. Cells were washed three times and incubated for 60 min at 4 °C with secondary antibodies (except for directly conjugated antibodies). After washing, the cells were analyzed using a flow cytometer (FACSCalibur; BD Biosciences). Negative control cultures were incubated with secondary antibodies alone. The experiments were performed in triplicate and the acquired data were as analyzed with Flowing software, version 2.5.1 (BD Biosciences).

### Transplantation of human NECs into chick embryo

Chicken fertilized eggs from Hy-Line W36 strain hens were incubated at 37.5 °C, 60% humidity, with turning by 90° every hour for 40–42 h. Chick embryos were used at stage HH11 according to Hamburger and Hamilton (1951). To manipulate chicken embryos, the surrogate egg shell system was used. Double yolk chicken eggshells were prepared as recipient of incubated embryos as previously described (Borwompinyo et al., 2005). To minimize bacterial and fungal contamination of cultures, all procedures were performed under laminar airflow hood after wiping eggs with 70% ethanol.

The blunt-end of the recipient egg was removed using a small hand-held electric drill to create a 42- to 45-mm window and the yolk and albumen was discarded. Next, the incubated eggs were opened at the pointed end using a small hand-held electric drill to create a 30- to 32-mm window and the embryo was gently transferred into the recipient eggs. Eggs were positioned with the window at the top to allow access to the embryo through the window. Embryos were counterstained *in ovo* with fast green dye (0.1% (w/v) solution in PBS) supplemented with penicillin–streptomycin, which was injected at a point outside the embryonic plate under the embryos using an insulin syringe needle which was bended at a 45-degree angle. Afterwards, the vitelline membrane was slit open over the anterior pole of the embryo with an insulin syringe needle and the selected neuroepithelial segment was excised from the anterior diencephalon. A correspondingly-sized piece of epithelium from the hPSC-NEC was cut with an insulin syringe needle, transferred to the host using the needle, and grafted into the chick embryo. The piece of donor tissue was implanted into the previously prepared site in the host. The NEC area was 300 ± 70 μm^2^ (n = 30) and the number of cells per transplantation was 7.2 × 10^5^ ± 1.1 × 10^5^ (n = 30). Eggs were sealed with a piece of cellophane wrap (Saran Wrap) along with thin albumen to adhere the wrap to the shell. The window was secured using a pair of plastic rings embedded with 4 screws with rubber bands extending from the rings. Operated embryos were returned to the incubator at 37.5 °C and 60% RH without titling for 14 h. Afterwards, the eggs were turned at an angle of 30° at hourly intervals until analysis of the chimeras. Embryonic mortality was monitored at days 5 and 14 and chimeric embryos were sacrificed at 19 days post-implantation or as indicated.

### MRI and histology

MRI was performed according to standard protocols with Siemens Prisma 3T. Histological analysis of tissues was performed on 6 µm tissue sections, sectioned with microtome (microm, Germany), according to standard protocols after fixation in 10% buffered formalin and staining with hematoxylin and eosin (H&E) and Nissl.

### Immunofluorescence staining of tissue sections

Tissues were fixed in 4% paraformaldehyde for 20 min at 4 °C followed by washing in PBS three times for 10 min. Tissues were allowed to sink in 30% sucrose overnight and then embedded in OCT (optimal cutting temperature compound, Bio Optica 05-9801) and cryosectioned at 20 mm. For immunofluorescence staining, sections were blocked and permeabilized in 0.1% Triton X-100% and 4% normal donkey or goat serum in PBS. Sections were then incubated with primary antibodies in 0.1% Triton X-100, 4% normal donkey serum.

The following primary antibodies were used: SOX2 (rabbit, Chemicon, AB5603, 1:500), RELN (mouse, Millipore MAB5366, 1:500), LMX1A (rabbit, Abcam, ab139726, 1:250), FGF8 (rabbit, Abcam, ab81384, 1:200), OTX2 (rabbit, Abcam, ab21990, 1:100), FOXA2 (rabbit, Abcam, ab40874, 1:100), GFP (mouse, Santa cruz, SC9996, 1:200), FGF18 (mouse, Santa cruz, SC393471 1:100), WNT3A (rabbit, Abcam, ab28472, 1:100), β-Catenin (rabbit, Abcam, ab6302, 1:200), BMP4 (rabbit, Abcam, ab3973, 1:200), STEM121 (mouse, Clontech, Y40410, 1:100), HNA (mouse, Chemicon, MAB1281, 1:200), GFAP (rabbit, Abcam, ab2760, 1:200), PAX2 (mouse, Thermo, H00005076, 1:200), and PH3 (mouse, Abcam, ab14955, 1:200).

Secondary antibodies were used goat, and donkey Alexa Fluor 488, 568 and 647 conjugates (Abcam, ab150105, 150108, ab150157, ab150107, 1:500).

Images were captured with Confocal microscopy (Zeiss LSM800) and analyzed with ImageJ software.

### Electrophysiological studies

After 17 days of transplantation, chicken embryos were removed from the egg. The iTeO was rapidly dissected and submerged in ice-cold cutting solution containing (in mM) 2.5 KCl, 0.5 CaCl_2_, 2 MgCl_2_, 1 NaH_2_PO_4_, 26.2 NaHCO_3_, 238 sucrose and 11 D-glucose bubbled with 95% O_2_- 5% CO_2_. Transverse slices (400 μm) were prepared using a vibrotome (VT1200S; Leica, Nussloch, Germany). Subsequently, slices were transferred to standard artificial cerebrospinal fluid (aCSF) containing (in mM) 125 NaCl, 3 KCl, 1.25 NaH_2_PO_4_, 25 NaHCO_3_, 10 D-Glucose, 2 CaCl_2_, 1.3 MgCl_2_ bubbled with 95% O_2_- 5% CO_2_. The osmolality of above-mentioned solutions was in the range of 295–300 mOsm and pH was adjusted to 7.3–7.4. Slices were then incubated for 20 min at 35 °C and stored at RT until they were individually transferred to a submerged recording chamber. The ecording chamber was mounted on a fixed-stage upright microscope (Axioskop 2 FS MOT; Carl Zeiss) and continually perfused at 2 mL/min with standard aCSF at RT. The iTeO layers and neurons were visualized using an IR-CCD camera (IR-1000, MTI) with a 10X or ×40 water immersion objective lenses.

Whole-cell voltage-clamp and current-clamp recordings were made using a Multiclamp 700B (Molecular Devices) equipped with Digidata 1440A analogue to digital interface (Molecular Devices) at RT. For voltage clamp recording, data were sampled at 20 kHz using a low-pass filter at 2 kHz. For current clamp recording, data were sampled at 10 kHz and low-pass filtered at 10 kHz. Data were recorded on a PC using pCLAMP 10 software (Molecular Devices). Patch pipettes were prepared from borosilicate glass capillaries (1.5 mm OD, 0.75 mm ID, Sutter Instruments) with a micropipette puller (P-97, Sutter Instruments) and had resistances of 3–5 MΩ. The pipette capacitance was compensated using the negative capacitance circuit of the amplifier. Series resistance was 10–20 MΩ and compensated by at least 70%.

For examination of electrophysiological properties of neurons and capability of action potential generation, the pipette solution contained (in mM): 115 K-gluconate, 20 KCl, 10 HEPES, 2 EGTA, 10 Na_2_-phosphocreatine, 2 MgATP and 0.3 Na_2_GTP. pH was adjusted to 7.25–7.30 and osmolality was in the range of 290–295 mOsm. Action potentials were evoked (in current clamp mode) by injecting depolarizing current pulses up to 400 pA in 100 pA steps. Membrane potential was held at −60 mV when resting membrane potential of neurons was more positive than −50 mV.

For recording the voltage-gated sodium and potassium currents, the micro-pipette was filled with a solution as for the action potential recording. CaCl_2_ in bathing solution was replaced with equimolar of cobalt chloride to avoid contamination with the calcium currents. Voltage was stepped from a holding potential of −70 mV to test potentials from −90 to +30 mV in 10 mV increments. Potassium currents were recorded in the presence of 1 µM tetrodotoxin (TTX, Tocris, 1078) in bathing solution. For analysis, maximal current amplitude was measured after leak subtraction.

Checking the synaptic current was performed in voltage clamp. For recording of ionotropic glutamatergic currents, micro-pipettes were filled with a solution contained (in mM): 102 CsMeSo_4_, 20 HEPES, 5 TEA-Cl, 11 BAPTA, 0.2 EGTA, 2 MgATP, 0.3 Na_2_GTP and 5 QX-314. pH was adjusted to 7.25–7.30 and osmolality was in the range of 290–295 mOsm. AMPA receptor-mediated currents were inspected at holding potential of −70 mV in the presence of bicuculline methiodide (20 μM, Sigma-Aldrich, 14343) and APV (50 μM, Sigma-Aldrich, A8054) in aCSF, and NMDA receptor-mediated currents were recorded at holding potential of −10 mV in the presence of bicuculline methiodide (20 μM, Sigma-Aldrich, 14,343) and CNQX (20 μM, Sigma-Aldrich, C127) in aCSF. For examination of GABA_A_ receptor-mediated currents, pipette solution contained (in mM): 140 CsCl, 2 MgCl_2_, 1 CaCl_2_, 10 HEPES,10 EGTA, 2 MgATP, 2 Na_2_GTP and 5 QX-314. Recordings were made at holding potential of −70 mV in the presence of CNQX (20 μM, Sigma-Aldrich, C127) and NMDA (50 μM, Sigma-Aldrich, A8054) in aCSF. Both spontaneous and evoked synaptic currents were checked.

### Transplantation of quail isthmic organizer

Fertile chick eggs were incubated for approximately 40 h to reach developmental stage HH11, after which chick embryos were accessed by windowing the eggs. To open the brain vesicle, an incision was made in the anterior diencephalic neuroepithelium. A portion of the isthmus from a stage-matched quail embryo was carefully isolated and grafted into the diencephalon of the host chick embryo.

Following the transplantation, the chick embryos were returned to incubation for an additional 10 days. After this period, chick foetuses were perfused with 4% paraformaldehyde (PFA) for tissue fixation. The brains were subsequently isolated from the cranial cavity, processed for histological analysis, and embedded in paraffin. Serial cross-sections were cut at a thickness of 10 µm.

To enable cellular and structural analysis, alternate sections were stained using Nissl staining and immunostained with an anti-quail antibody (QCPN, obtained from the Hybridoma Bank, Iowa) to specifically label quail-derived cells.

### RNA extraction from tissues and cells and library preparation for RNA-sequencing

Tissue samples were homogenized using the BeadBug microtube homogenizer (Biozyme) and subsequently passed through QIAshredder homogenizing column (Qiagen) followed by RNA extraction with miRNeasy micro kit (Qiagen). Cell pellets were lysed and homogenized by Qiazol incubation followed by QIAshredder column and RNA isolation by miRNeasy micro kit. Libraries for cell and tissue samples were generated by SMARTer Stranded Total RNA Sample Prep Kit - HI Mammalian (Clontech).

### Bioinformatics and statistical analyses

Sequencing was performed on NextSeq500 platform (Illumina) using v2 chemistry, resulting in over 20 M reads per library with 75bp single end setup. The resulting raw reads were assessed for quality and adapter content with FastQC (Andrews, 2010). Trimmomatic (Bolger et al., 2014) was employed to trim reads ends where average base calling qualities (in 5 bp windows) were below Q20. Only reads with minimum length of 30 bp were kept for further analyses. When working with tissue RNA-sequencing data, to separate reads that originated from human and chicken cells, we concatenated Ensembl human genome version hg38 (GRCh38) and Ensembl chicken genome version galgal5 (5.0.90), created an index file for this “chimeric reference genome”, and aligned all trimmed and filtered reads into it by using STAR version 2.7.1a (Dobin et al., 2013). Then, uniquely mapped reads were counted using featureCounts in Subread toolkit 1.6.4 (Liao et al., 2014). By this approach, each read was only aligned and counted for the reference genome that had higher similarity to, and the reads with equal similarity to both genomes were excluded. Counts normalization and differential expression analysis were performed using the R/Bioconductor package DESeq2 (Love et al., 2014). Genes having absolute log_2_ fold-change higher than 1 and Benjamini–Hochberg adjusted *P-*value less than 0.05 were considered as significant differentially expressed genes (DEGs). The shrinkage of log fold changes was performed using “ashr” shrinkage estimator (Stephens, 2017). Visualization of the results was done by ggplot2, pheatmap and EnhancedVolcano packages. Overrepresentation analysis was performed in DAVID Bioinformatics Resources (Sherman et al., 2022). Gene set enrichment analysis was performed using Broad Institute GSEA software (Subramanian et al., 2005).

## Results

### Implantation of hPSC-NECs induces an ectopic optic tectum in the diencephalon of chicken embryos

**FIGURE 1:**
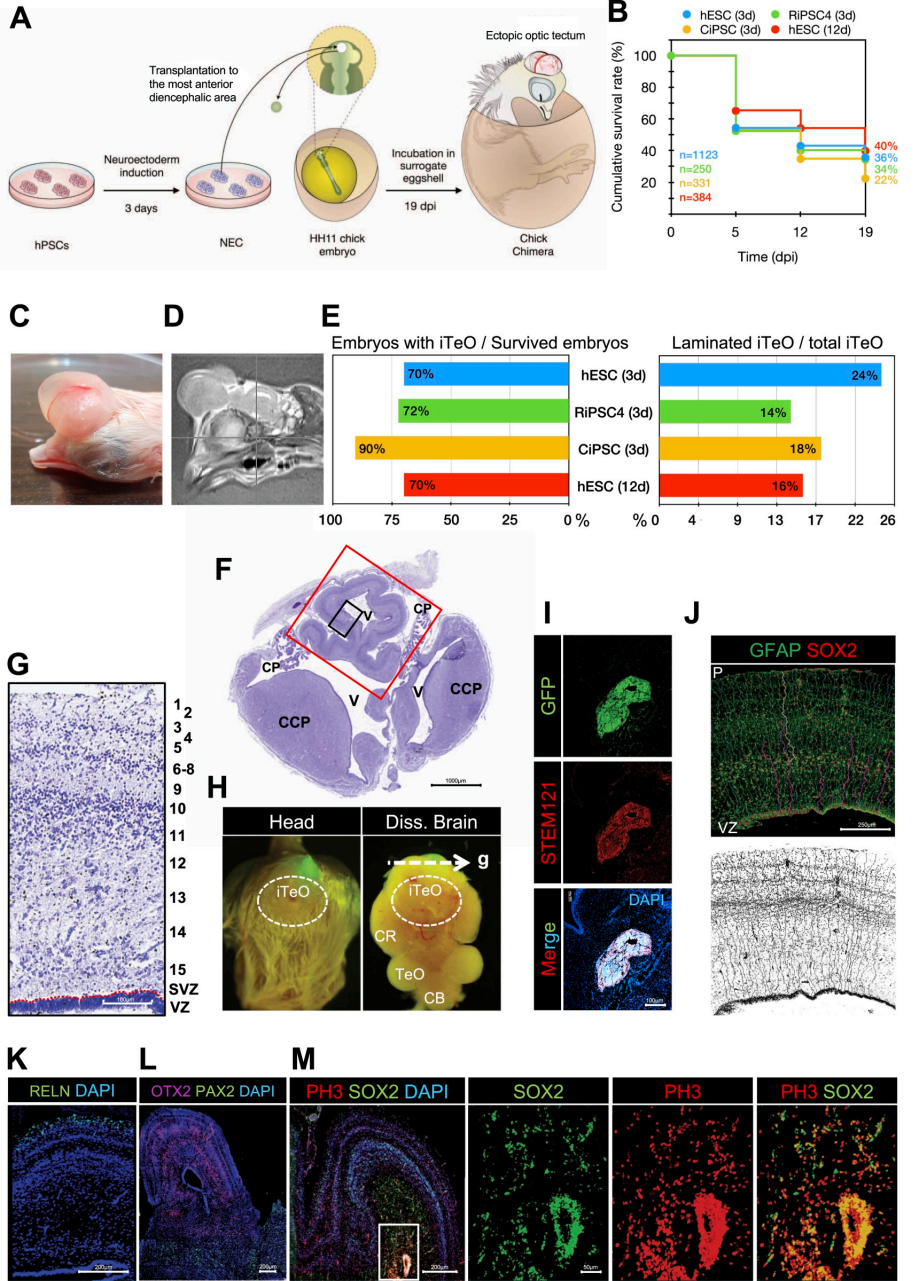
Intradiencephalic grafting of hPSC-NECs into chick embryos and characterization of induced optic tectum 19 days after implantation. **(A)** Schematic figure depicting the strategy for generation of the human cells-chick xenograft. Neuroepithelial cells (NECs) were differentiated from either hESCs or hiPSCs and implanted in chick embryos (stage 11 HH) by replacing a similarly sized part of the diencephalon. **(B)** Kaplan–Meier survival curve of chick embryos after engraftment with NECs which generated by the 3-day or 12-day protocol from different hPSC lines. **(C)** Macroscopic images of an ectopic tissue or induced optic tectum (iTeO). **(D)** Magnetic resonance imaging (MRI) of an iTeO at a mid-sagittal plane. **(E)** Comparison of iTeO induction percentage rate between different cell lines and protocols (left panel) and laminated iTeO to total iTeO ratio (right panel). **(F)** Whole-mount coronal section of a chicken brain that grafted with hPSC-NECs after Nissl staining and harvested at 19-day post-implantation (dpi); the ectopic brain part is outlined in red inset. The black inset exhibiting stereotypical stratified layers parallel to the brain surface. Bona fide folding of the layered iTeO is apparent, including folding of the pial surface and the underlying neuronal layers, while the ventricular surface remains smooth. **(G)** Higher magnification of bright-field image after Nissl staining (black inset of f), showing layers of iTeO. The layers were numbered based on Ramón y Cajal classification of chicken natural optic tectum (TeO) (Wylie et al., 2009). The dotted red line indicates the VZ/SVZ border. **(H)** Chicken brain, grafted with green fluorescent protein (GFP)-labelled hPSC-NECs at 19 dpi. The ectopic brain part is outlined by a white dotted circle. The right panel shows an image of the dissected brain including the iTeO. The position of the coronal section of GFP-positive cells shown in I is indicated. CR: Cerebrum, TeO: Optic tectum, CB: Cerebellum. **(I)** Immunofluorescence of coronal sections with localized expression of GFP and after staining with human cell specific antibody (STEM121) to demonstrate presence of human cells. Nuclei were counterstained with DAPI (blue). **(J)** Immunofluorescence staining of coronal sections for GFAP and SOX2, showing arrangement of apical redial glial cells (aRGCs) at the luminal side of ventricle zone (VZ) of iTeO. The aRGC fiber scaffold is oriented radially and elongated in the iTeO. The black and white picture in bottom panel demonstrates elongated aRGCs in iTeO. P: Pial side. **(K)** Immunofluorescence staining for RELN in the iTeO, close to the pial surface. **(L)** Immunofluorescence staining for OTX2 and PAX2 in the iTeO. **(M)** Immunofluorescence staining for phospho-histone H3 (PH3) and SOX2 to mark proliferating neural progenitor cells in iTeO. Nuclei were counterstained with DAPI (blue).

To evaluate whether hPSCs can be induced to form neuroepithelial secondary organizers, we differentiated one hESC line (RH6 line, with or without green fluorescent protein (GFP)) and two hiPSC lines (RiPS4 and CiPS lines) into NECs, followed by *in ovo* cross-species transplantation into the anterior diencephalon of early chick embryos. Rapid and efficient induction of NECs was achieved by exposure of adherent hPSCs to a cocktail of small molecules and growth factors (Supplementary Figure S1A). Essentially, we concomitantly inhibited SMADs and modulated the retinoic acid, WNT, and SHH pathways, which are known to direct neuroepithelial development during embryogenesis *in vivo* (Chambers et al., 2009; Tabar and Studer, 2014; Pasca et al., 2015; Suzuki and Vanderhaeghen, 2015; Tchieu et al., 2017). Acquisition of NEC identity occurred after only 3 days as indicated by rosette morphology and near homogeneous expression of neural progenitor-specific markers (Supplementary Figures S1B-S1E). We also generated NECs (12d-NEC) using a published protocol, which requires 12 days to reach nearly homogeneous expression of neural progenitor-specific markers (Tchieu et al., 2017); (Supplementary Figures S1E, F). Sheets of NECs derived from hESCs (3d or 12d protocol, hESC-3d or -12d NECs) or hiPSCs (3d protocol, hiPSC-3d-NECs) were cut by a needle under a stereomicroscope before intra-neural tube implantation into a hole made in the diencephalon of early stage (HH11) chicken embryos (Figure 1A; Supplementary Figures S2A-C).

Approximately 22%–40% of 2088 hPSC-NEC grafted embryos survived up to 19-day post-implantation (dpi) and were used for further analysis (Figure 1B). Stereomicroscopical inspection revealed that 70%–90% of surviving embryos developed ectopic structures in the diencephalon (Figures 1C–E).

Further histological analysis of ectopic tissues using cryo- and paraffin sections indicated that implantation of hESC-3d-NECs induced formation of an architecture composed of different layers of neurons, strongly resembling the morphology of the TeO in chicken embryos (Figures 1F,G; Supplementary Figures S2D,E). Thus, we named the newly emerged architectures “induced optic tectum” (iTeO). Neurons within iTeOs were tangentially arranged and formed stratified layers parallel to the brain surface (Figures 1F,G). 14%–24% of iTeOs were laminated at 19 dpi with a thickness of 740.6 ± 128.6 µm (Figure 1E). Other ectopic structures were not laminated and not further followed up. Laminated iTeO with similar morphology were reproducibly generated from hiPSC-3d-NECs (Supplementary Figures S3A–C) and hESC-12d-NECs (Supplementary Figures S3D–F). Interestingly, several laminated iTeO underwent folding of the pial surface and the underlying neuronal layers, whereas the ventricular surface remained straight (Figure 1F; Supplementary Figure S3B).

Notably, transplantation of NECs into the spinal cord (n = 64, of which 39% survived until 19 dpi) and chorioallantoic membrane (n = 51, of which 61% survived until 19 dpi) did not result in iTeO formation or any other type of host tissue reorganization. Instead, transplanted NECs formed neural rosettes when placed directly adjacent to the spinal cord as reported before (Goldstein et al., 2002). In agreement with previous reports (Hidalgo-Sanchez et al., 2022), transplantation of quail donor isthmic organizers into the anterior diencephalon of chick embryos at HH11 did not induce tectum-like lamination of the host tissue (n = 9, Supplementary Figure S2F). We concluded that the interactions between hPSC-derived neuroepithelial cells and the diencephalon of the host differ substantially from the interaction of the quail IsO with the host tissue. Such differences may account for the ability of hPSC-derived neuroepithelial cells but not the quail IsO to initiate structural features of tectum in the anterior diencephalon at HH11 stage.

To trace transplanted human cells in chicken brains, hESCs were labelled with GFP using a CMV early enhancer/chicken beta actin promoter-GFP construct (CAG-hrGFP hESC) (Figure 1H). Staining with a GFP antibody, a human cell specific antibody (STEM121) and/or a human nuclear antigen (HNA) antibody revealed that transplanted cells essentially did not migrate into the laminated area of the chicken iTeO up to 19 dpi (0.001%, HNA+/DAPI) (Figure 1I; Supplementary Figures S2G, H), indicating that laminated iTeOs are derived from chicken host cells.

Parallel elongation of radial glial cells (RGCs) provides a scaffold for neuronal migration, eventually giving rise to the columnar distribution of pyramidal neurons (Gray and Sanes, 1992; Lever et al., 2014). To explore whether apical radial glial cells (aRGCs) acquire a distinct identity during induction of lamina formation, we stained aRGCs in iTeO for GFAP (Figure 1J). Interestingly, GFAP-immunoreactive aRGC fibers showed a radial orientation and elongated in iTeO (Figure 1J). Reelin expression was found in the uppermost layer of iTeOs, suggesting ongoing neuronal migration and axon guidance (Candal et al., 2005; Di Donato et al., 2018) (Figure 1K), whereas expression of OTX2 and PAX2 confirmed differentiation of tectal neurons within iTeOs (Nakamura, 2001) (Figure 1L). Despite expression of midbrain markers, we did not observe expression of hindbrain marker TSHZ2 in the induced structures (Supplementary Figure S2I).

Formation of the iTeO in the chicken diencephalon was accompanied by cell proliferation in the subventricular zone (SVZ), indicated by the presence of double-positive SOX2+ (neural progenitor marker) and PH3+ (G2/M phase marker phosphohistone-3) cells (Figure 1M). We also observed expression of HOPX, another neural progenitor marker, in SOX2+ cells (Supplementary Figure S2J). These findings suggest increased proliferative activity in the SVZ, probably to cope with the increased demand for neurons to generate iTeOs.

We next examined electrical features of neurons in the iTeOs. Voltage clamp recordings from individual cells of 1-9 layers revealed the presence of voltage-gated sodium currents blocked by tetrodotoxin (TTX, 1 μM), and voltage-gated potassium currents blocked by 4-aminopyridine (4-AP, 5 mM) and tetraethylammonium (TEA, 10 mM) (Supplementary Figure S4A). Electrophysiological recordings revealed that iTeOs neurons developed action potentials in response to square depolarizing currents (from 50 to 200 pA in 50 pA increment and duration of 600 ms) (Supplementary Figure S4B). The frequency of action potentials increased when depolarizing currents were enhanced (Supplementary Figure S4C). To determine the action potential threshold in neurons of different layers, a ramp depolarizing current was applied. Rheobase current was ranged from 10.99 to 192.50 pA among the neurons (Supplementary Figure S4D). Electrophysiological analysis confirmed synaptic activity in developing iTeOs, suggesting functionality of the iTeOs neurons (Supplementary Figures S4E–I).

### Gene expression in iTeOs resembles gene expression in the normal optic tectum, reflecting generation of electrically active neurons

Next, we compared patterns of gene expression in the iTeO, the TeO and the natural chicken cerebral cortex (NCC) through RNA sequencing analysis. RNA sequencing reads were mapped to a chicken-human chimeric genome, revealing that over 99.6% of uniquely mapped and assigned reads correspond to the chicken cells. This finding confirmed results from the cell tracing analysis, indicating that very few if any of the transplanted human NECs migrate into the iTeO.

**FIGURE 2:**
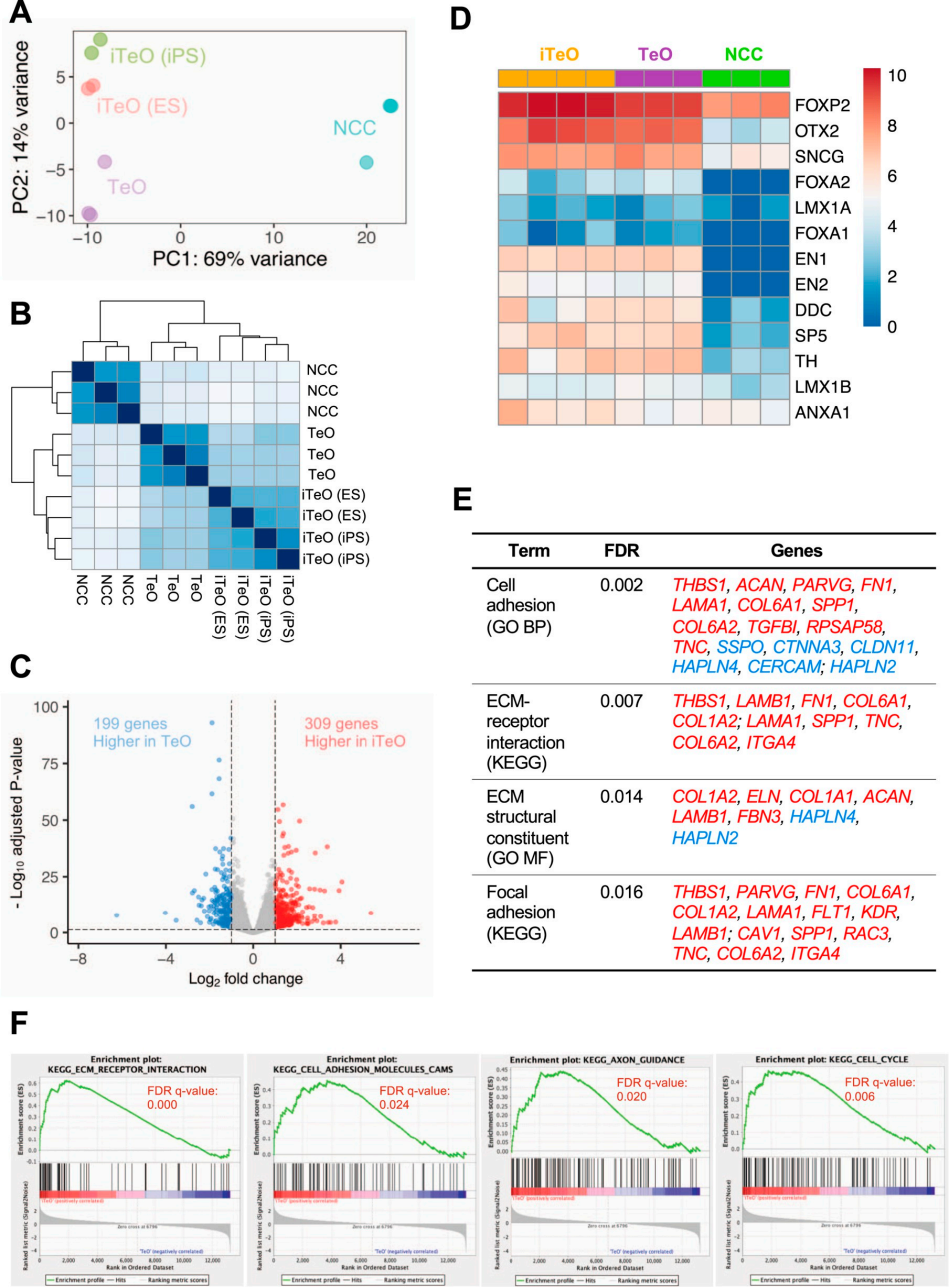
Gene expression profiles of iTeO and TeO. **(A)** Principal component analysis (PCA) plot based on transcriptome profiles of hESC (ES)-3d- and hiPSC4 (iPS)-3d-iTeO, normal TeO and chicken cerebrum (NCC). **(B)** Heatmap of samples distance matrix comparing iTeO (ES), iTeO (iPS), TeO and NCC based on transcriptome profiles. **(C)** Volcano plot showing differential gene expression between hPSC-iTeO and TeO. Red dots indicate genes significantly more expressed in iTeO, whereas blue dots refer to those with significantly higher expression in TeO. Genes with adjusted P-value <0.05 and shrunken absolute log_2_ fold change >1 were considered as significantly different in expression. **(D)** Heatmap of midbrain markers in iTeO, TeO and NCC. The color bar shows log2-normalized counts. **(E)** Representative GO and KEGG pathway biological terms enriched (with FDR <0.1) in genes differentially expressed between iTeO and TeO. Red indicates significantly upregulated in iTeO, whereas blue refers to those significantly upregulated in TeO. **(F)** Gene set enrichment analysis (GSEA) in iTeO vs. TeO. Gene sets were derived from MSigDB hallmark and KEGG pathway gene sets.

Principal component analysis (PCA) showed that cells in the iTeO are more similar to cells in the TeO than to NCC. Only minor differences were observed between iTeOs induced by hESC-NECs and iTeOs induced by hiPSC-NECs (Figure 2A). Analysis of correlation heatmaps confirmed the close relation between iTeOs and TeOs and their dissimilarity to NCC (Figure 2B). The search for differentially expressed genes (DEGs) between iTeO and TeO uncovered 508 significant DEGs (adjusted p-value <0.05 and absolute shrunken log_2_ fold change >1). 309 genes were significantly higher expressed in iTeO, whereas 199 genes were significantly higher in TeO (Figure 2C). In contrast, iTeO and NCC showed 2011 significant DEGs. Differences between iTeOs and TeOs were comparatively low. Relaxation of the absolute shrunken log_2_ fold change cutoff to 2 revealed only 62 DEGs. Of note, iTeO and TeO showed a similar expression of midbrain markers, which differed from the transcriptional profile of NCCs (Figure 2D). To better characterize genes that are differently expressed between iTeO versus TeO, we performed an overrepresentation analysis using DAVID on DEGs (adjusted p-value <0.05 and absolute shrunken log_2_ fold change >1). Only a few terms were significantly enriched (FDR <0.1) in iTeO vs. TeO DEGs when using the GO biological process, GO molecular function and KEGG pathway databases. Terms included cell adhesion, ECM-receptor interaction, extracellular matrix structural constituent and focal adhesion, all of which contribute to brain and TeO development (Figure 2E). Interestingly, expression of DCX, a marker for neuronal migration and immature neurons in early stages of chicken TeO development (Lever et al., 2014), was almost 3-fold higher in iTeO compared to TeO (adjusted P-value = 3.67E-17). Similarly, gene set enrichment analysis (GSEA) indicated significant enrichment of gene sets in iTeO related to ECM-receptor interaction, cell adhesion molecules, proliferation of progenitors, and axon guidance (Figure 2F). Such gene sets are indicative for neural migration in the developing brain and TeO and for axon guidance. Taken together, these results suggest that hPSC- NECs induce an ectopic TeO when transplanted into the anterior diencephalon of chicken embryos, which resembles the natural TeO by morphology and gene expression. Differences between iTeO and TeO are mainly caused by different developmental states, as identified by differential expression of general neurodevelopment-related genes, rather than by differences in cell identity, i.e., key tissue markers.

### *In vitro* differentiated human NECs acquire organizing center potential after transplantation into the chicken diencephalon

**FIGURE 3:**
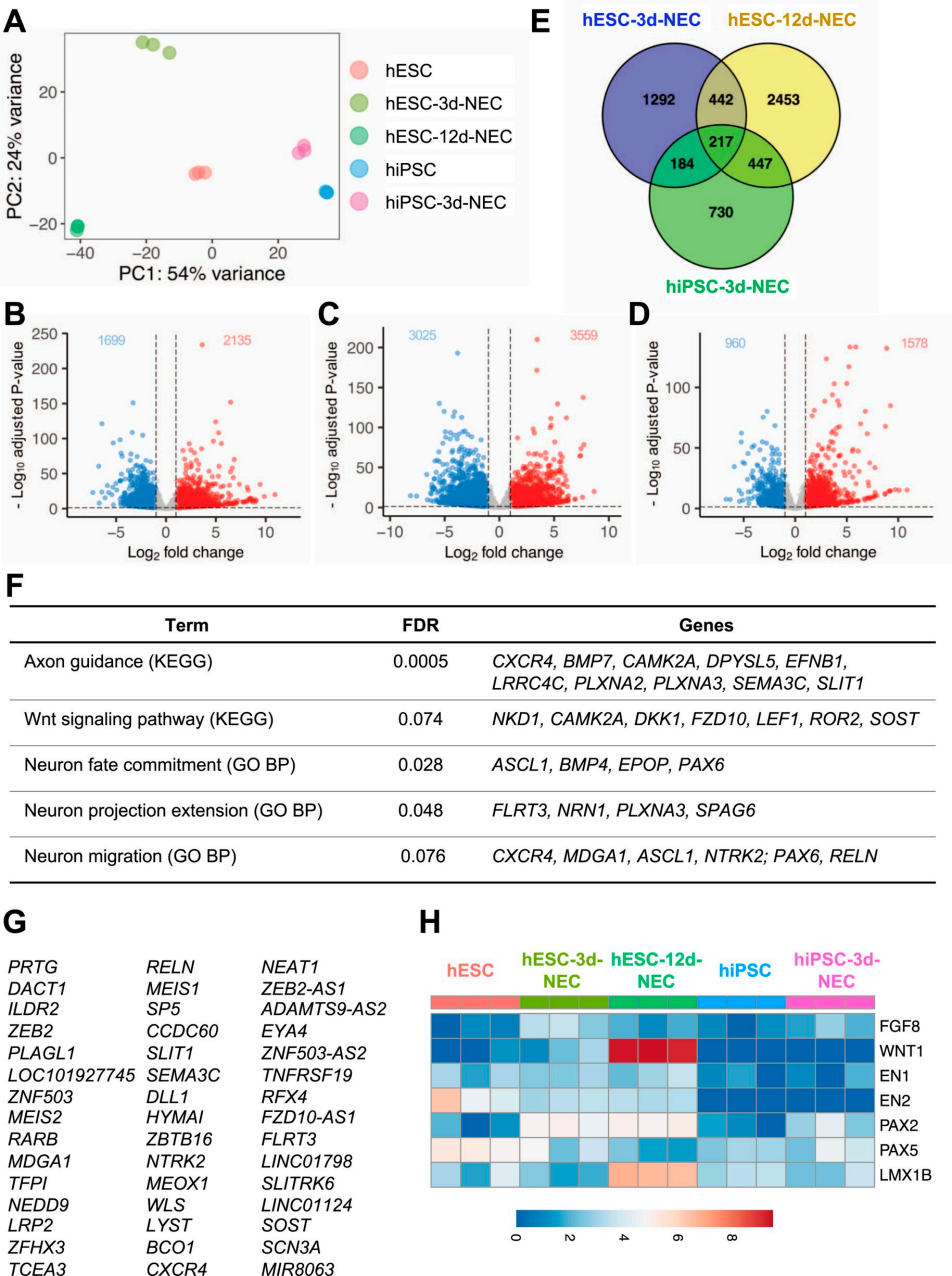
Transcriptome analysis of NECs derived from different hPSCs. **(A)** Principal component analysis (PCA) plot based on transcriptome profiles of hPSCs (hESC and hiPSC) and NECs generated using the 3d or 12d protocol. **(B–D)** Volcano plot representation of changes in gene expression profiles between hPSCs and NECs. **(B)** hESC-3d-NEC vs. hESC, **(C)** hESC-12d-NEC vs. hESC, **(D)** hiPSC-3d-NEC vs. hiPSC (hiPSC4). Red and blue dots indicate genes significantly up- and downregulated in NECs compared with hPSCs, respectively. The number of each category is indicated. **(E)** Venn diagram representation of the overlap between significantly upregulated genes in NECs (derived from different hPSCs using different differentiation protocols) compared with hPSCs. **(F)** Enrichment of representative biological terms (FDR <0.1) in upregulated genes of NECs. **(G)** List of significantly and highly upregulated genes (adjusted P-value <0.05 and Log2-fold change >2) in NECs derived from different hPSCs using different differentiation protocols. **(H)** Heatmap representation of expression of isthmic organizer (IsO) markers in hPSCs and hPSCs-NECs. The color bar shows log2-normalized counts.

To explore how hPSC-NECs acquire secondary organizer properties that can induce development of the chicken TeO, we characterized transcriptional changes during differentiation of hPSCs into NECs (Figure 3). By using RNA-sequencing, we analyzed the transcriptome profiles of hPSCs and NECs (Figure 3A; Supplementary Figure S5A). Concomitant downregulation of pluripotency markers and upregulation of neuroepithelial markers suggested successful differentiation of hPSCs towards NECs (Supplementary Figure S5B). Next, we compared hESC-3d-NEC and hESC-12d-NEC with hESC and hiPSC-3d-NEC with hiPSC (Figures 3B–D).

Overrepresentation analysis of upregulated genes (adjusted P-value <0.05 and Log_2_ fold change >1) in differentiating hESC-3d-NECs, hESC-12d-NECs, and hiPSC-3d-NECs (Figure 3E), revealed significant enrichment of biological terms such as axon guidance, Wnt signaling pathway, neuron fate commitment, and neuron migration (Figure 3F). Robustly upregulated genes (adjusted P-value <0.05 and Log_2_ fold change >2) (Figure 3G) were also mainly involved generally in brain development and axon guidance. Of note, FGF8 which is required for induction and function of the IsO, was not among the strongly upregulated genes in human NECs after *in vitro* differentiation. Essentially, the NECs did not show a full IsO identity *in vitro*, despite some changes in the expression of IsO markers including *FGF8*, *WNT1*, *EN1*, *EN2*, *PAX2*, *PAX5,* and *LMX1B* during *in vitro* differentiation (Figure 3H). We reasoned that human NECs either acquire full IsO qualities only after interaction with chicken host tissue or induce formation of an IsO within the chicken diencephalon.

**FIGURE 4:**
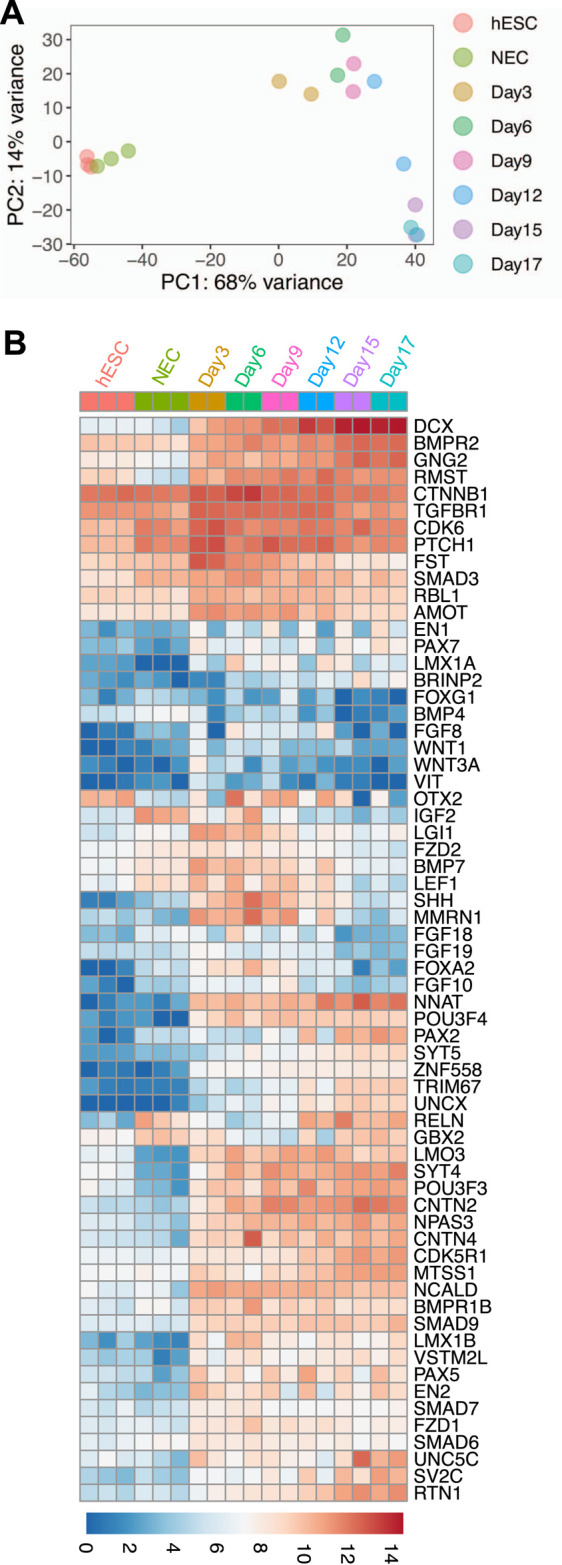
Analysis of temporal changes in the transcriptome of transplanted human cells. **(A)** Principal component analysis (PCA) plot based on transcriptome profiles of human ESCs, after *in vitro* differentiation to NECs and at different time points after transplantation of NECs into chicken embryo hosts. NEC were generated from hESCs using the 3d protocol (hESC-3d-NEC). **(B)** Heatmap of gene expression in hESCs, hESC-3d-NEC and transplanted NECs at different time points. The color bar represents log2-normalized counts.

To distinguish between these possibilities, we analyzed gene expression within the hESC-3d-NECs after transplantation. Human GFP-positive NECs which are located within the rostral to dorsal midline of iTeOs (Supplementary Figure S6) were mechanically dissected along with neighboring chicken cells under a fluorescence stereomicroscope, 3, 6, 9, 12, 15 and 17 days (D3-D17) after implantation. RNA-seq data obtained from isolated samples were subjected to a bioinformatics pipeline, allowing discrimination between human and chicken transcripts and quantification of temporal expression changes (Figure 4A; Supplementary Figure S7A). Before transplantation, we did not find noteworthy expression of the IsO marker FGF8, the Anterior Neural Ridge (ANR) marker FOXG1, and the Zona Limitans Intrathalamica (ZLI) marker SHH in NECs (Figure 4B). We observed upregulation of several signaling molecules including those involved in WNT, BMP, TGF-β, and SHH signaling pathways in human NECs after transplantation, which may at least in part account for induction of the TeO (Figure 4B). The role of these signaling pathways in proliferation of the TeO cells and neurogenesis has been previously reported (Roussa et al., 2006; Ille et al., 2007; Cole et al., 2016; Shitasako et al., 2017). The alteration in SHH signaling components expression during iTeO development is most likely connected to the massive expansion of TeO cells (Agarwala et al., 2001; Dahmane et al., 2001; Feijoo et al., 2011; Rapacioli et al., 2012).

**FIGURE 5:**
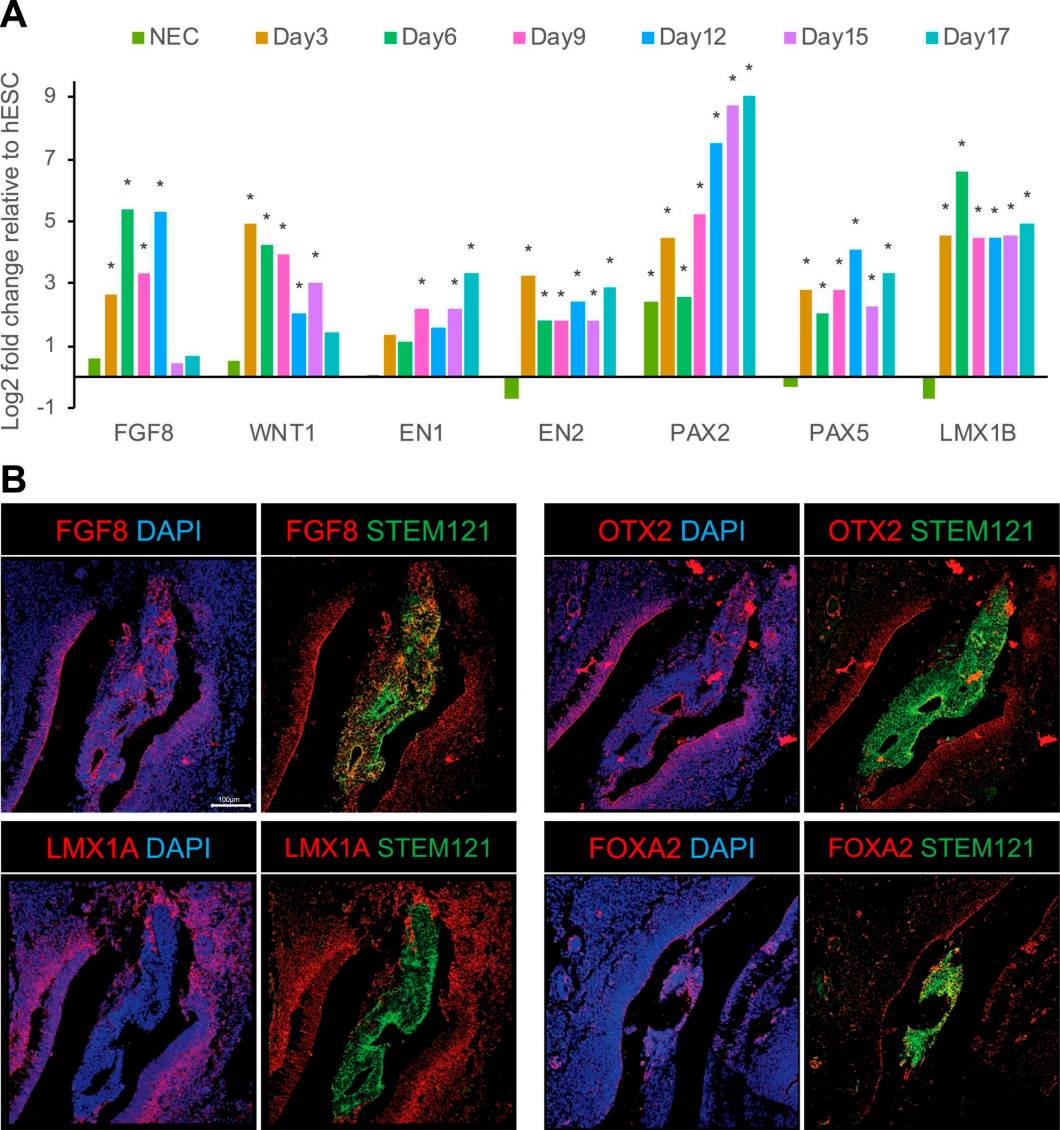
Grafted human NECs acquire characteristics of the isthmic organizer. **(A)** Log2-fold expression changes of isthmic organizer (IsO) markers genes in NEC (generated by the 3d protocol) before and at different timepoints after transplantation relative to hESC obtained from transcriptome analysis. Values were derived from data obtained in RNA-seq experiments. Shrunken Log_2_ fold changes relative to hESC and Benjamini–Hochberg adjusted *P-*values for indicated genes were extracted from differential gene expression analysis performed using DESeq2. Asterisks indicate an adjusted P-value <0.05. **(B)** Immunofluorescence analysis of sections for different IsO-related markers (FGF8, OTX2, LMX1A, FOXA2) at 6 dpi (days post-implantation). Staining for a human-specific antigen STEM121 was used to identify transplanted hPSC-NECs. Nuclei were counterstained with DAPI (blue).

**FIGURE 6:**
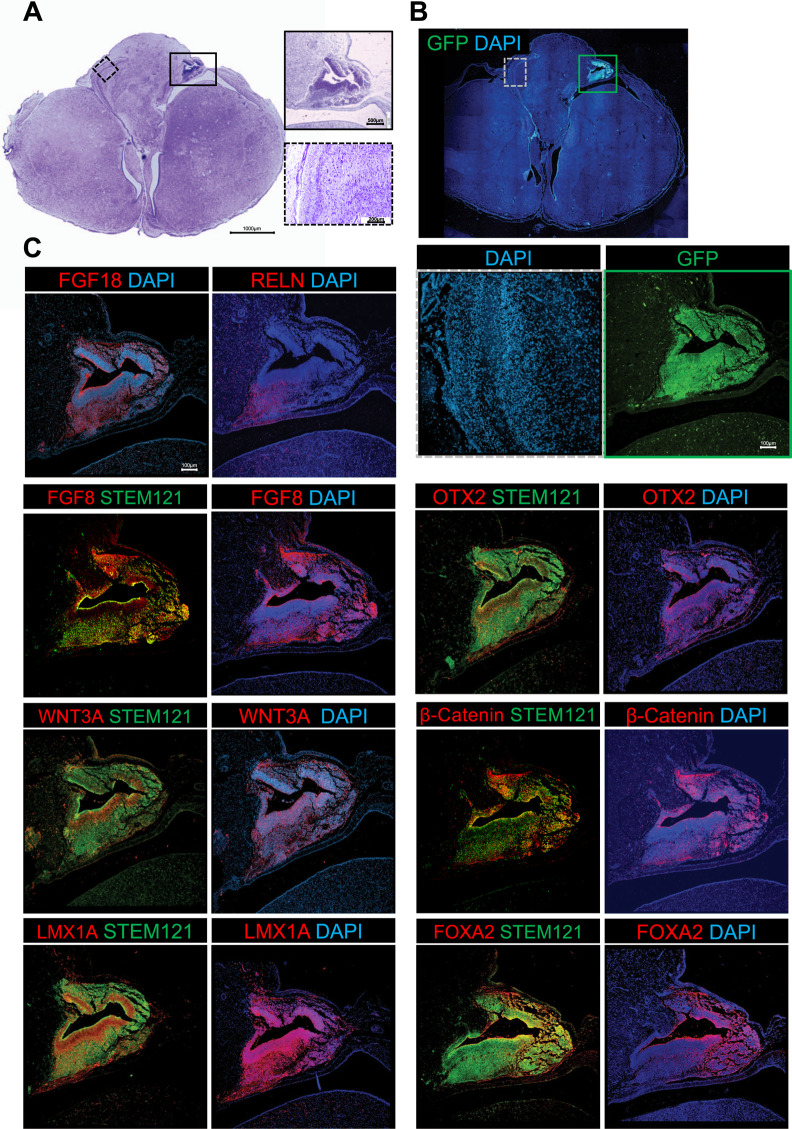
Expression of isthmic organizer markers in transplanted human cells. **(A)** Coronal sections of chicken induced optic tectum (iTeO) grafted with hESC-3d-NECs and harvested at 19 dpi after Nissl staining. Boxed areas on the left encompassing the grafted area and the induced stratified layers are shown at higher magnification on the right. **(B)** Immunofluorescence image depicting the area of GFP expression, containing transplanted hPSC-NECs at 19 dpi. Coronal sections were immunostained with a GFP antibody and analyzed using confocal microscopy. The dotted box delineates the laminated area without GFP positive cells. Nuclei were counterstained with DAPI (blue). **(C)** Immunofluorescence staining of coronal sections for the isthmic organizer (IsO)-related markers FGF18, FGF8, OTX2, WNT3A, β-catenin, LMX1A, and FOAXA2 in transplanted human GFP-positive cells. Immunofluorescence for the human-specific antigen STEM121 was used to identify transplanted hPSC-NECs. Nuclei were counterstained with DAPI (blue).

Most importantly, we observed a significant increase in expression of IsO markers in transplanted, but not in non-transplanted NECs relative to hESCs, including FGF8, which is a crucial molecule for development of the IsO and mandatory for induction of the TeO (Figure 5A). In addition, we detected a strong protein expression of FGF8 in transplanted human cells at Day 6 and Day 19, which were identified by GFP expression and staining with human cell specific antibody STEM121 (Figures 5B, 6). Expression of FGF8 was complemented by the appearance of other secreted factors directing formation and lamination of the TeO, such as FGF18, WNT3A, and Reelin (Figures 5B, 6). Expression of FGF8 and lack of FOXG1 expression (Figure 4B) in transplanted cells argues against the possibility of other secondary organizers, i.e., ZLI and ANR formation. Since robust expression of IsO markers in NECs occurred only after transplantation, we concluded that NECs receive signals from the adjacent host neuroepithelium, which induces maturation of human NECs into *bona fide* IsO cells. This hypothesis is supported by expression of OTX2, LMX1A and FOXA2 in the transplants, which maintain IsO identity (Figures 5B, 6).

The distinct IsO-type expression profile that emerged in human NECs after transplantation strongly suggests that the transplanted human cells acquire IsO-properties, sufficient to induce TeO formation. However, it also is possible that neighboring chicken cells are recruited into the IsO and contribute to its activity. To investigate this possibility, we analyzed chicken host cells surrounding the transplanted human cells, which indeed expressed several signaling molecules including *FGF8* at Day3 (Supplementary Figure S7B). In addition, these cells showed early expression of *EYA1* and *SIX1* genes, which control *FGF8* expression (Guo et al., 2011). The dynamic expression changes in the host tissue, elicited by the transplantation of NECs, may also explain why only human NECs but not regular quail IsO were able to induce TeO formation at a rather late developmental stage (HH11). At present, it is difficult to distinguish whether chicken or human cells first express FGF8, since expression in either cell type occurred simultaneously and FGF8 is well known to induce its own expression when ectopically applied to the neuroepithelium (Martinez et al., 1999). However, the chicken host cells surrounding the transplanted human cells did not robustly express other IsO markers despite expression of *FGF8*, (Supplementary Figure S7B), suggesting that recruited chicken cells do not acquire full IsO properties. Taken together, our results suggest that human NECs mature into an IsO-like secondary organizer after transplantation into the diencephalon of developing chicken embryo, which eventually results into induction of an ectopic TeO.

## Discussion

Here, we demonstrated that hPSC-NECs induce formation of an ectopic TeO after transplantation into the most anterior diencephalic region of chicken embryo. This observation came as a surprise, since we initially wanted to investigate whether human NECs are able to develop into orthotopic structure of the chicken brain. hPSC-NECs start to express some IsO markers during *in vitro* differentiation but only acquire a broader set of IsO markers after interaction with the host environment. These results indicate that hPSC-NECs are receptive to instructional cues from adjacent embryonic host tissue, demonstrating the potential of hPSCs to model complex morphogenetic events. We reason that the hPSC-NEC-chicken embryo xenotransplantation system will facilitate better understanding of the mechanisms of midbrain development, including analysis of involved signalling pathways, specification of cell types, and lamination of induced tissue.

Examination of the layered architecture revealed extension of RGCs from the apical to the basal side, although clear lamination was observed only in about 20% of iTeOs. RGCs, which play a fundamental role in orchestrating brain development and TeO lamination, guide migration of newborn neurons and first appear at embryonic day 6 in the chicken TeO. Between embryonic days E12 and E20 they transform into astrocytes, complementing tangential migration of nascent neurons (Lever et al., 2014; Watanabe and Yaginuma, 2015). The transplanted hPSC-NECs as well as the developing iTeO express Reelin, which is instrumental to regulate extension and orientation of radial fibers (Hartfuss et al., 2003; Zhao et al., 2004; Nomura et al., 2008). Suppression of Reelin expression prevents ordered projection of radial fibers in the dentate granule cell layer and formation of laminar architectures is incapacitated (Weiss et al., 2003; Zhao et al., 2004). Engraftment of Reelin-expressing COS7 cells into the marginal zone of the quail embryonic cortex increases extensions of aRGCs and their attachment to the pia, but did not modify the cortical architecture (Nomura et al., 2008). The morphological changes induced by transplantation of hPSC-NECs go far beyond extension of aRGCs, indicating that Reelin expression is only one of several hallmarks of hPSC-NECs. Transplantation of hPSC-derived NECs also resulted in increased proliferation of neural stem and/or progenitor cells in SVZ resulting in increased folding of the iTeO, a phenomenon that was also observed by overexpression of FGF2 or Thymosin B4 in the developing chicken TeO (McGowan et al., 2012; Wirsching et al., 2012).

Transcriptome analysis of iTeOs revealed a high similarity to the natural TeO and clear differences to the NCC, excluding the possibility that the layered architecture that forms after transplantation of hPSC-NECs represents mammalian cerebral cortex. Differences between experimentally iTeO and naturally emerging TeO are mainly related to genes involved in ECM-receptor interactions, cell adhesion, and cell cycle, which play fundamental roles in neural cell migration, axon guidance, neural network formation and ultimately brain and TeO development (de Curtis, 1991; Reichardt and Tomaselli, 1991; Letourneau et al., 1994; Yamagata et al., 1995; Miskevich et al., 1998; Myers et al., 2011; Yang et al., 2019), suggesting that iTeOs are at an earlier developmental stage compared to TeOs. Induction of ECM genes in developing iTeO might be caused by release of TGF-β signalling molecules or other factors from transplanted human cells (Verrecchia and Mauviel, 2002; Deng et al., 2024). The upregulation of ECM genes may play a fundamental role in formation of the observed SVZ niche in the iTeO, as reported before (Kerever and Arikawa-Hirasawa, 2021).

Our results demonstrate that transplanted hPSC-NECs acquire some properties of the IsO-like cells and develop further after transplantation, allowing induction of the chicken TeO. Previous studies indicated that native IsO tissue grafts transplanted into the brain of avian embryo change the fate of surrounding tissues to mesencephalon and metencephalon (Wurst and Bally-Cuif, 2001). However, when we transplanted a quail donor isthmic organizer into the anterior diencephalon of chick embryos at HH11 stage, we did not observe an induction of the optic tectum in the host tissue, suggesting differences between the quail IsO and hPSC-NECs, which is in line with our previous observations. hPSC-NECs only acquire IsO properties after transplantation, indicating complex mutual interactions between the host and the grafted tissue. The observation that transplanted cells induce optic tectum in the host suggests acquisition of a genuine isthmic organizer identity through host interactions, rather than just reflecting a temporal delay, although we cannot rule out that differences in developmental timing between human and chick cells play a role. We assume that molecules secreted by hPSC-NECs alter the responsiveness of the host tissue, while acquiring IsO functions on their part, eventually empowering transplanted cells to induce an optic tectum at this region and developmental stage. Furthermore, we cannot exclude that species-specific or other differences between the artificial, hPSC-NEC-derived IsO and the natural quail IsO account for the different responsiveness of the chicken diencephalon to inductive cues. Earlier attempts to generate human IsO-like cells *in vitro* relied on modulation of the WNT signalling pathway using the small molecule CHIR99021. Such IsO-like cells produced FGF8 and WNT1 accompanied by upregulation of IsO markers, but functionality for mid-hind brain induction was not evaluated (Lee et al., 2018). We conclude that hPSC-NECs act as *bona fide* IsOs and not the chicken cells that surround transplanted human cells, since these cells only exhibit early FGF8 expression but not the full spectrum of IsO markers. The absence of other IsO markers except FGF8 in chicken cells adjacent to the NEC transplants was surprising, since FGF8 is able to form an IsO-like center in the embryonic chicken forebrain (Crossley et al., 1996; Irving and Mason, 2000). FGF8 signalling specifies the midbrain by inducing expression of EN1 and PAX2 in OTX2-expressing region (Harada et al., 2016), which is consistent with our results showing expression of PAX2 and OTX2 in developing iTeOs. In addition to FGF8, as the main signalling molecule for IsO formation and function, we observed an increase in the expression of WNT1 and LMX1B in human NECs after transplantation. Which are also important for the organizer formation and maintenance (Adams et al., 2000; Canning et al., 2007; Guo et al., 2007; Rapacioli et al., 2016). Development of transplanted NECs into IsO and not ZLI and ANR secondary organizers probably depends on transplantation site, stage and *in vitro* differentiation protocol. Further, in contrast to the surrounding chicken cells, hPSC-NECs show robust expression of signalling molecules and intracellular factors with possible roles in maintaining identity of the organizer including FGF18 (Liu et al., 2003; Sato et al., 2004), WNT3A (Buckles et al., 2004), B-Catenin (Chilov et al., 2010), OTX2 (Tour et al., 2002), LMX1A (Chung et al., 2009) and FOXA2 (Chung et al., 2009). We speculate that the formation of a strong IsO in hPSC-NECs induces expression of FGF8 in adjacent chicken cells but otherwise blocks further maturation into an IsO. Further experiments are needed to validate this hypothesis.

In conclusion, we have developed a model to study induction of tectum formation by generating IsO-like cells from hPSCs. Since it is straightforward to introduce loss- or gain-of-function mutations into hPSC-NECs, we will be able to model human brain development and disorders involving defects of the IsO and tectum malformations. Thus, the combination of hPSC differentiation *in vitro* and transplantation *in ovo* will help to further identify and characterize processes critical for the pathogenesis of human brain disorders.

## Data Availability

The RNA-seq data in this study have been submitted to the NCBI Gene Expression Omnibus (GEO; https://www.ncbi.nlm.nih.gov/geo) under the accession number GSE235580.
