# Supplementary material for: Human pluripotent stem cell-derived neuroepithelial cells develop into an organizer for optic tectum formation in the chicken diencephalon
Source: Front Cell Dev Biol. 2026 Jun 1;14:1812038. doi: 10.3389/fcell.2026.1812038 (PMC13265535; doi:10.3389/fcell.2026.1812038)
Supplement: Supplementary file 1 [file DataSheet1.pdf]

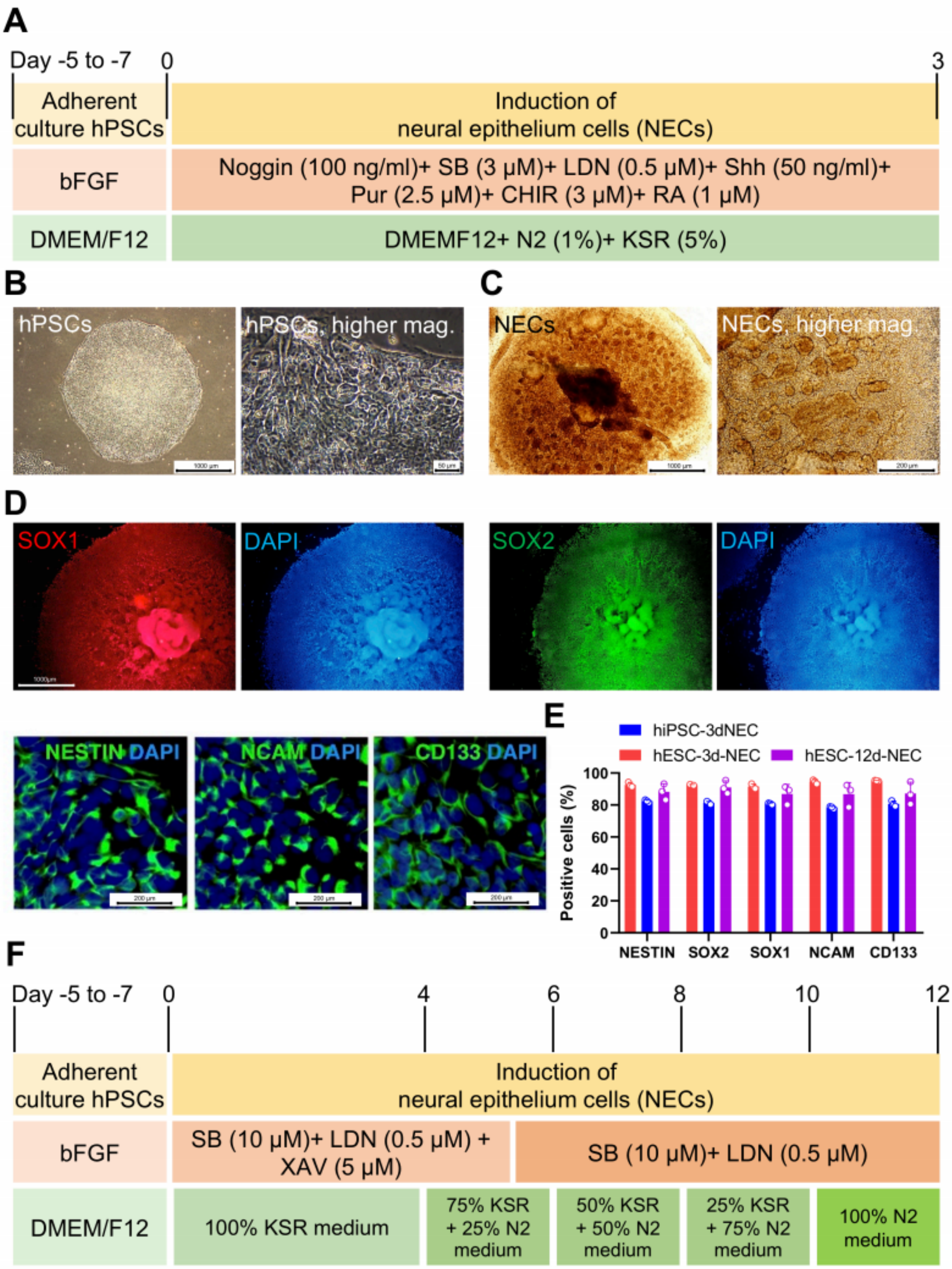

Figure S1. Generation of NECs from hPSCs. (A) Depiction of the 3-day protocol for differentiation of hPSCs into NECs. basic fibroblast growth factor (bFGF), Knockout serum replacement (KSR), noggin (a BMP inhibitor), sonic hedgehog (Shh), SB431542 (SB, TGFβ inhibitor), LDN193189 (LDN, a BMP inhibitor), purmorphamine (Pur, a Shh agonist), CHIR99021 (CHIR, a GSK3β inhibitor), and retinoic acid (RA). **(B)** Phase contrast images of hPSCs (RH6, hESCs). **(C)** Phase contrast images of NECs generated from hPSCs (RH6, hESCs). **(D)** Immunofluorescence staining of NECs for the neural progenitor markers SOX1, SOX2, NESTIN, NCAM, and CD133. Nuclei were counterstained with DAPI (blue). **(E)** Percentage of hESC-NECs, expressing neural progenitor markers. The 3-day and 12-day protocols were used for hESC-NECs and the 3-day protocol for hiPSCs (RiPSC4). **(F)** Depiction of the 12-day protocol for differentiation of hPSCs into NECs., basic fibroblast growth factor (bFGF), Knockout serum replacement (KSR), noggin (a BMP inhibitor), SB431542 (SB, TGFβ inhibitor), LDN193189 (LDN, a BMP inhibitor), XAV939 (XAV, a WNT pathway inhibitor).

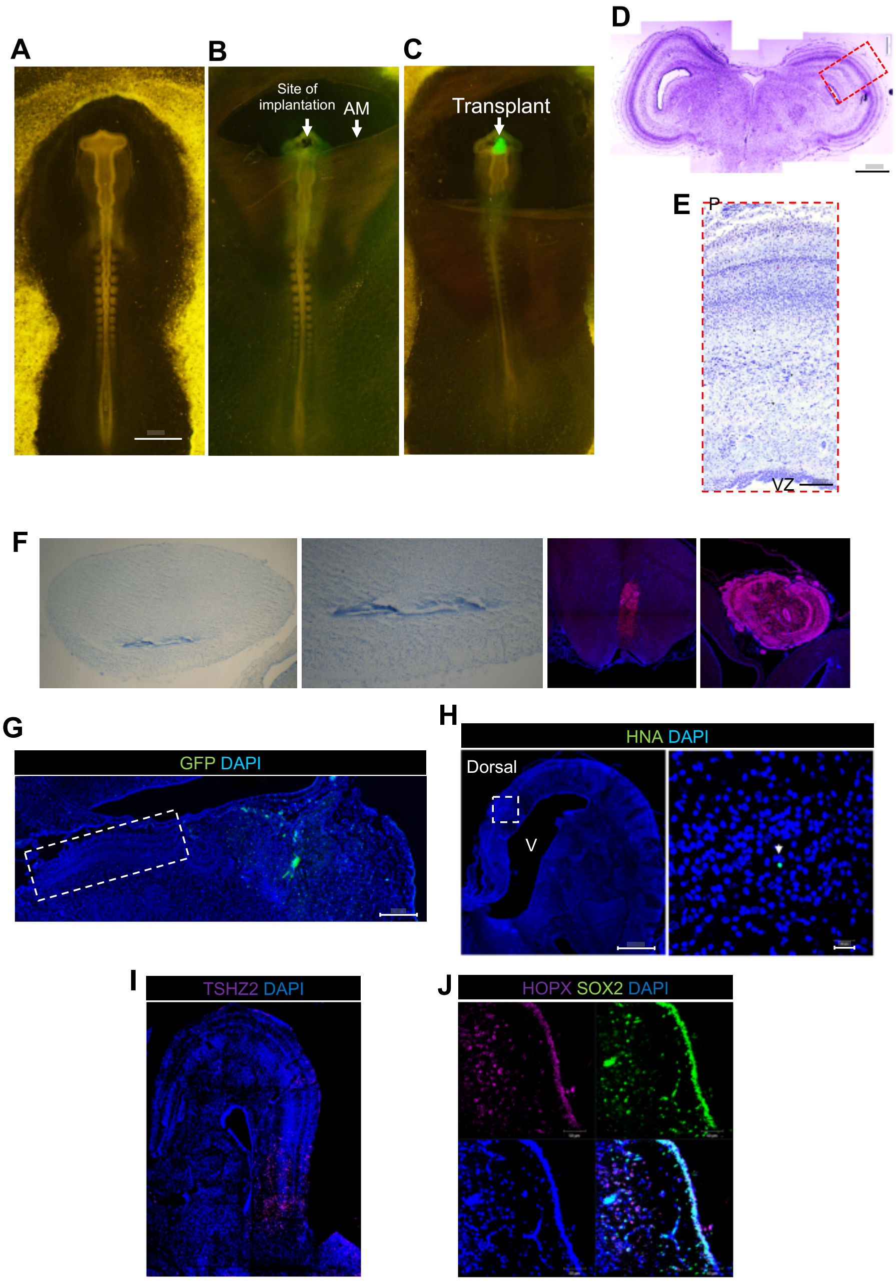

Figure S2. Induced and natural chicken optic tectum (iTeO *vs.* TeO). (A,B,C) Intradiencephalic implantation procedure of hPSC-NECs into chicken embryos. Whole-mount fluorescence images of chicken embryos at HH11 before manipulation (**A**), after removal of a piece of the diencephalon (**B**), and after implantation of GFP-labelled hPSC-NECs (**C**). Chicken embryo were visualized by sub-blastoderm injection of fast green. AM: amniotic membrane. **(D)** Coronal section of a normal chicken TeO after Nissl staining. **(E)** Higher magnification of bright-field image after Nissl staining (dotted red inset), showing layers of the natural chicken TeO. **(F)** Histological examination of the diencephalon from chick host fetuses transplanted by quail isthmus at HH11. While the grafted quail tissue is identifiable, the diencephalon lacks a laminated architecture in the region of the graft. **(G)** Tracing of human cells after transplantation. GFP^{+} NECs were grafted into chicken brain and harvested at 19 dpi. Coronal sections were immunostained with a GFP antibody and analyzed using confocal microscopy. The dotted box indicates the laminated area without GFP^{+} cells. **(H)** Immunofluorescence staining for human nuclear antigen (HNA), indicating that transplanted cells do not migrate into the laminated area of the chicken iTeO (0.001%, HNA+/DAPI). A higher magnification of the inset in the left image is shown in the right panel. **(I)** Immunofluorescence staining for TSHZ2 in the iTeO. **(J)** Immunofluorescence staining for HOPX and SOX2 to mark neural progenitor cells in iTeO. Nuclei were counterstained with DAPI.

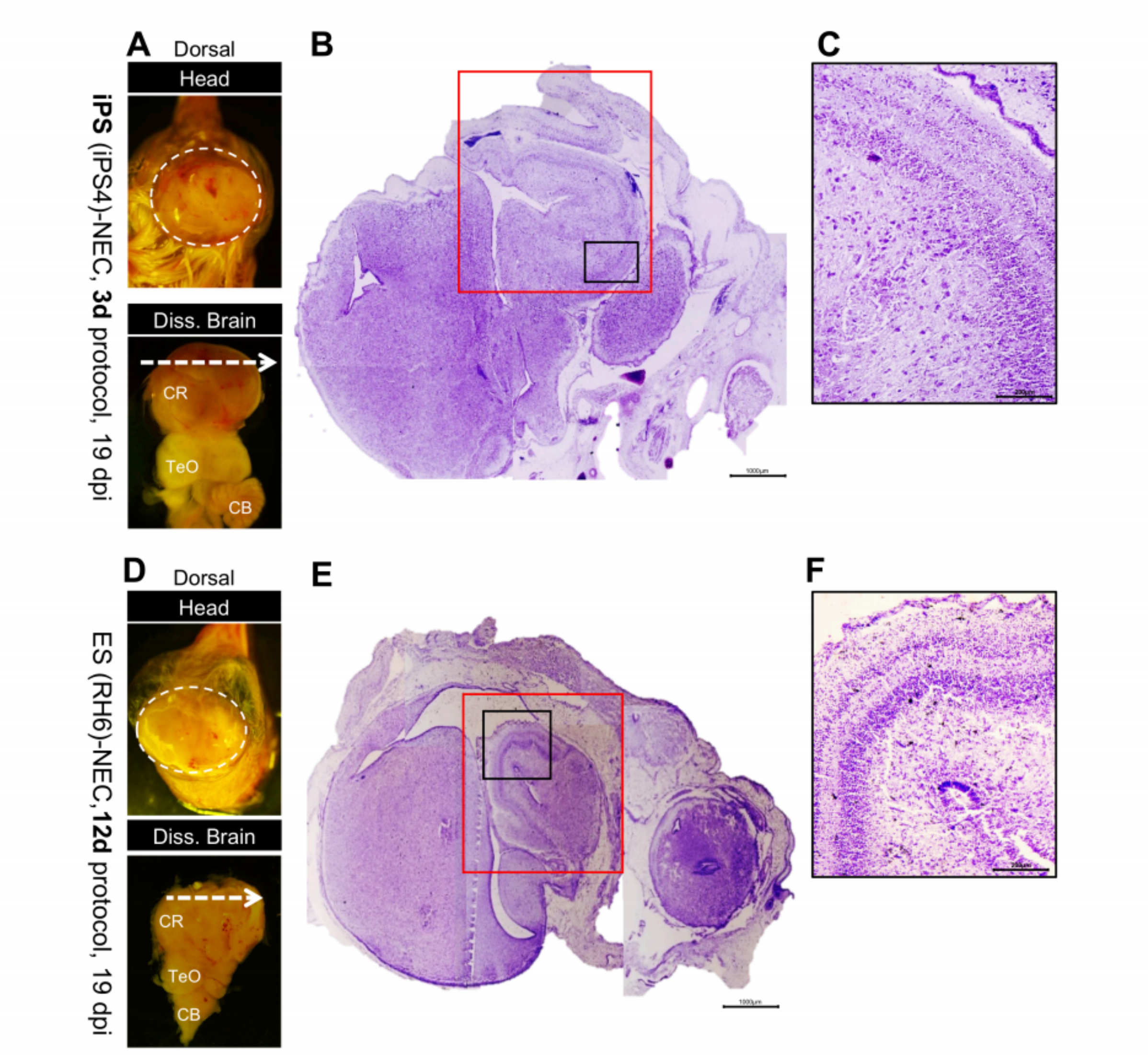

Figure S3. Morphology of iTeOs derived from hPSC-NECs. (A,D) Dorsal views of chicken heads after engraftment of hiPSC (RhiPSC4)-3d-NECs (**A**) or hESC (RH6)-12d-NECs (**D**), 19 days after implantation. iTeOs are labelled by a dotted white line. CR: Cerebrum, TeO: Optic tectum, CB: Cerebellum **(B,E)** Images of Nissl-stained hemisphere sections of chicken iTeO (red inset), revealing orientation of different tectal layers parallel to the brain surface (black inset) and folding of layered iTeOs. **(C,F)** High magnification images of Nissl-stained section of layered iTeOs.

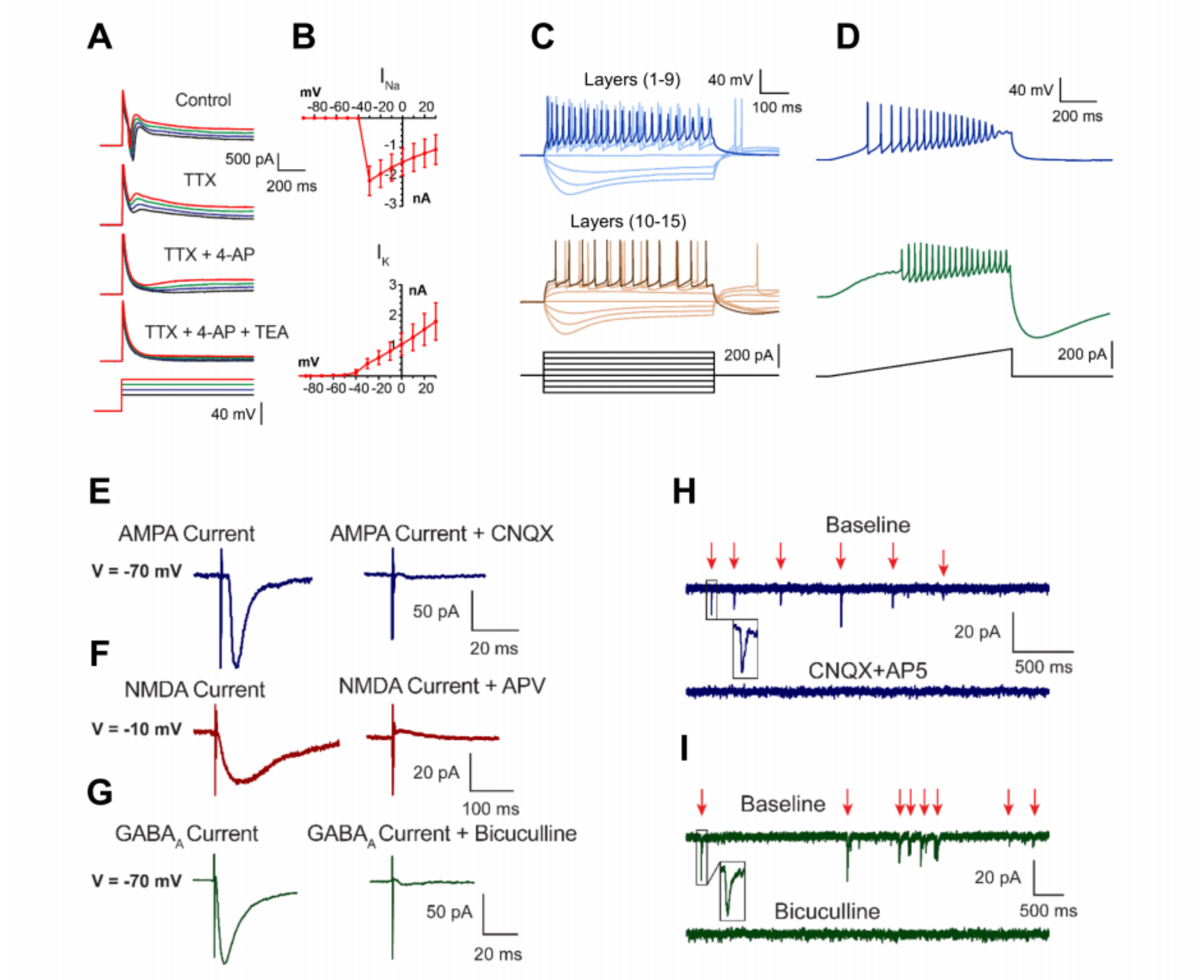

Figure S4. Electrophysiology properties and synaptic activity in whole-cell recordings of iTeO neurons. (A) Sample traces of voltage-dependent Na^+ and K^+ currents recorded from an iTeO neuron in layer (10-15) when the membrane potential was stepped from the holding potential of -90 mV (to relieve inactivation) to 0 - +30 mV in 10 mV increments. Application of TTX (1 μM) blocked the inward component demonstrating existence of voltage-activated Na^+ currents. Application of 4-AP (5 mM) and TEA (10 mM) removed fast and slow components of outward current demonstrating presence of voltage-activated K^+ currents. **(B)** Current-voltage curves of Na^+ and K^+ currents, plotted according to the amplitude of inward and outward currents respectively (n=5 cells from 3 iTeOs). **(C)** Single-neuron recordings of electrophysiological properties in 1-9 and 10-15 layers of iTeO by stepwise current injection (-150 to +200 pA, 600 ms, 50 pA increments, n = 25 for 1-9 layers and 18 for 10-15 layers). **(D)** Examples of neuronal firing of iTeO neurons in 1-9 and 10-15 layers in response to injection of depolarizing ramp current (200 pA, 1000 ms) to evaluate rheobase current, which was lower than 200 pA in 36 out of 39 cells. Evoked and spontaneous glutamatergic and GABAergic currents were recorded in the lower border of 1-9 layers. In iTeOs, spontaneous AMPA currents were observed in 6 out of 12 neurons and spontaneous $GABA_A$ currents were found in 10 out of 13 cells (each from 3 iTeOs). **(E)** Sample trace of evoked AMPA receptor current recorded at holding potential of -70 mV, without and with blockage by CNQX (20 μM). For recording the AMPA currents, 6 electrical pulses (240-650 μA; at 15 s interval) were applied through a metal electrode placed in 30-80 μm vertically far from recording cell in the lower border of 10-15 layers. Currents (167.73±16.81 pA) were obtained when the membrane potential was clamped at -70 mV in the presence of bicuculline (20 μM) and APV (50 μM) and were completely blocked by CNQX (20 μM). **(F)** Example of NMDA receptor current evoked at a holding potential of -10 mV. The current was eliminated by APV (50 μM). The evoked NMDA currents (22.14±5.44 pA) were recorded in the similar manner as AMPA currents except that the membrane potential was clamped at -10 mV and CNQX (20 μM) was applied instead of APV. Administration of APV (50 μM) to aCSF entirely removed the NMDA currents. In 5 out of 6 cells spontaneous

glutamatergic currents (16.26±2.87 pA) were also recorded, which were eliminated by applying both APV (50 µM) and CNQX (20 µM). **(G)** Sample record of evoked $GABA_A$ receptor mediated current at a holding potential of -70 mV, without and with inhibition by bicuculline (20 µM). The GABAergic currents were evoked at a membrane potential of -70 mV in the presence of CNQX (20 µM) and APV (50 µM) in aCSF. The stimulating electrode was placed in a similar position as for glutamatergic currents. **(H)** Recording of a spontaneous glutamatergic current at membrane potential of -10 mV. The red arrows show the spontaneous excitatory post-synaptic currents, which were blocked by CNQX (20 µM) and APV (50 µM). **(I)** Example of spontaneous currents mediated by $GABA_A$ receptors at a membrane potential of -70 mV. The red arrows indicate inhibitory post-synaptic currents that were inhibited by bicuculline (20 µM). Spontaneous GABAergic currents were recorded in 3 out of 3 cells. Both evoked (170.93±61.25 pA) and spontaneous (19.60±7.36) GABAergic currents were blocked by bicuculline (20 µM).

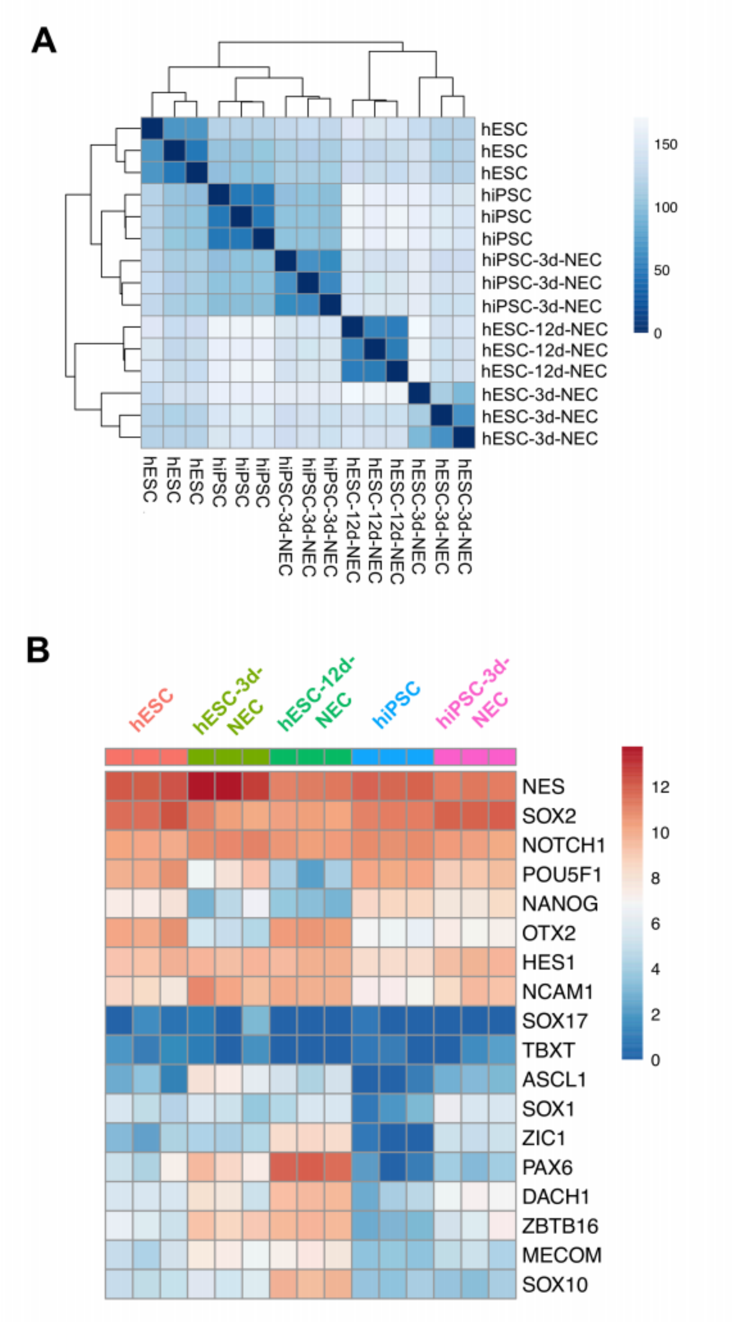

Figure S5. Transcriptome analysis of hPSCs in vitro differentiation toward NECs. (A) Correlation matrix comparing the distance between hPSCs and hPSCs-derived NECs based on transcriptome profiles. **(B)** Heatmap of pluripotency and neuroepithelial markers in hPSCs and hPSCs-NECs. The color bar represents log2-normalized counts.

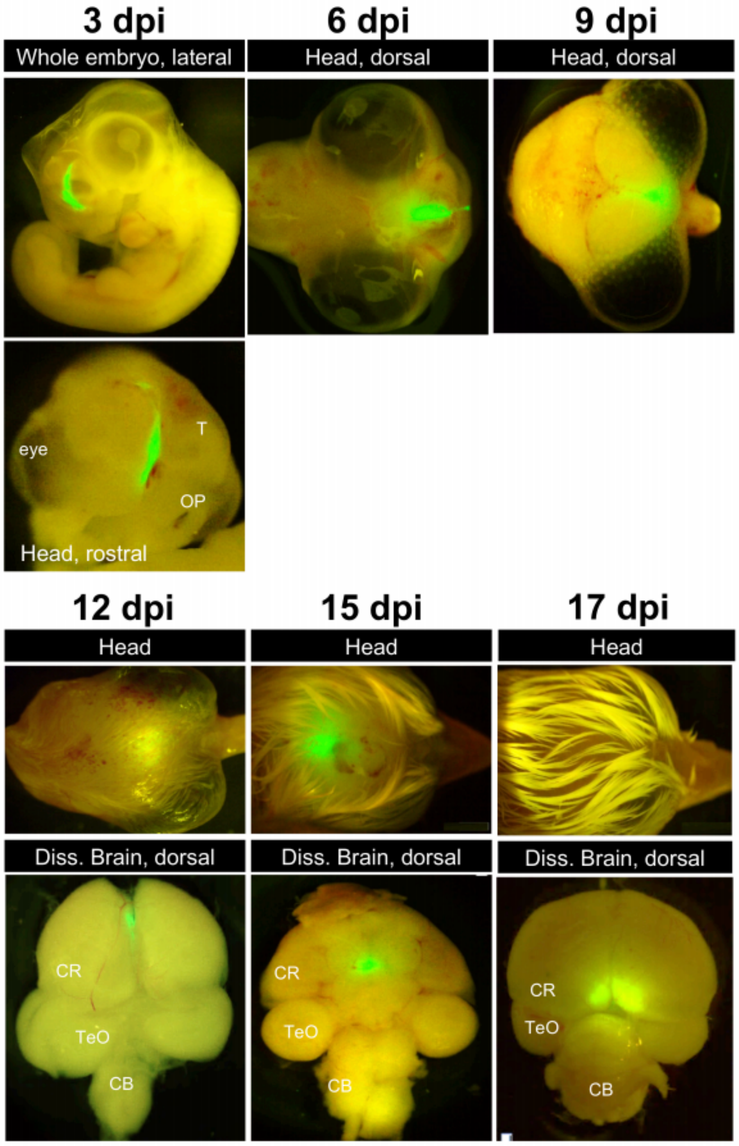

Figure S6. Temporal and spatial tracing of transplanted human cells. Whole-mount dorsal views of chicken embryos, heads, and dissected brains under fluorescence illumination after transplantation of GFP-labelled hPSC-derived NECs. GFP-positive cells are visible in the midline between both hemispheres and elongate up to the dorsal side. Top panel: NEC-implanted chicken embryos 3, 6, and 9 days after implantation (dpi). Bottom panel: NEC-implanted chicken embryos 12, 15, and 17 days after implantation. For further analysis, the GFP-positive areas were dissected under a fluorescence stereo microscope with a fine untoothed eye surgery forceps. OP: olfactory pits, T: telencephalon, TeO: optic tectum.

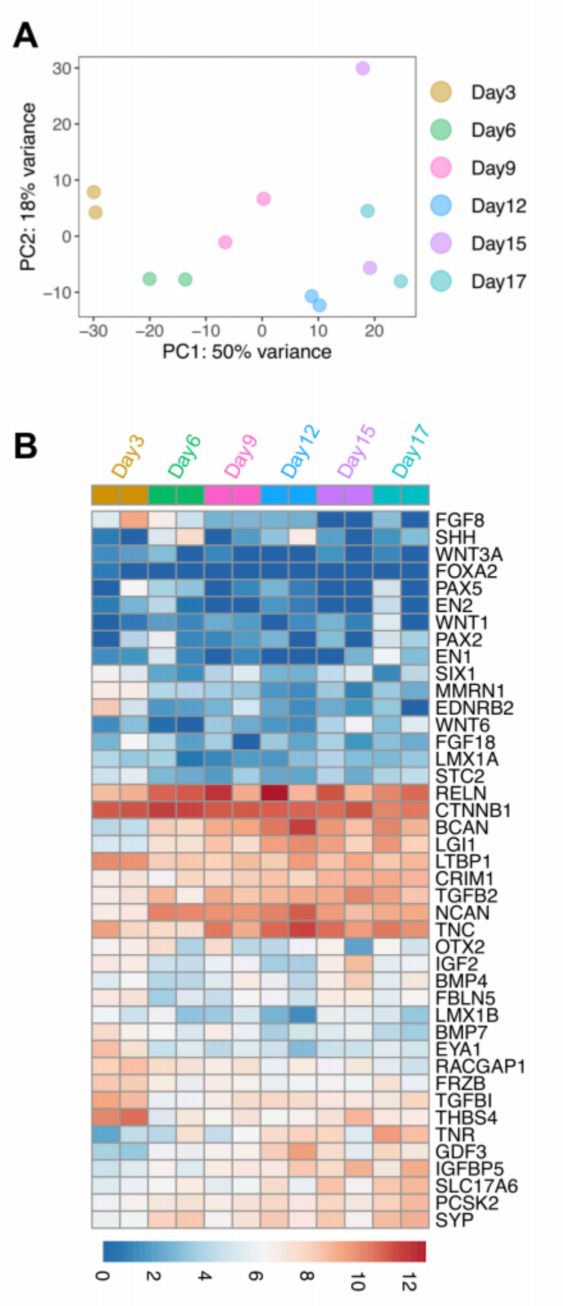

Figure S7. Transcriptional changes in chicken cells neighboring transplanted human cells over time. (A) Principal component analysis (PCA) plot derived from transcriptome profiles of chicken cells surrounding transplanted human cells at different time points. Chicken cells, neighboring human GFP-positive NECs, were mechanically dissected under a fluorescence stereomicroscope at different days post-implantation. **(B)** Heatmap of genes expression in chicken cells surrounding transplanted human GFP-positive NECs at different timepoints. The color bar represents log2-normalized counts.
